# Analysis of Atangana–Baleanu fractional-order SEAIR epidemic model with optimal control

**DOI:** 10.1186/s13662-021-03334-8

**Published:** 2021-03-19

**Authors:** Chernet Tuge Deressa, Gemechis File Duressa

**Affiliations:** Department of Mathematics, College of Natural Sciences, Jimma University, Jimma, Ethiopia

**Keywords:** SEAIR model, Atangana–Baleanu fractional derivative, Basic reproductive number, Global stability, Numerical simulation, Optimal control analysis

## Abstract

We consider a SEAIR epidemic model with Atangana–Baleanu fractional-order derivative. We approximate the solution of the model using the numerical scheme developed by Toufic and Atangana. The numerical simulation corresponding to several fractional orders shows that, as the fractional order reduces from 1, the spread of the endemic grows slower. Optimal control analysis and simulation show that the control strategy designed is operative in reducing the number of cases in different compartments. Moreover, simulating the optimal profile revealed that reducing the fractional-order from 1 leads to the need for quick starting of the application of the designed control strategy at the maximum possible level and maintaining it for the majority of the period of the pandemic.

## Introduction

Epidemiological mathematical models provide several aspects for understanding the dynamics of the spread of an epidemic and suggestions of effective control strategies. The insight that the transmission dynamics of endemic diseases can be formulated using mathematical language dates back to 1766 when Daniel Bernoulli published a paper where he described the effects of smallpox variolation on life expectancy [1]. Other early mathematical models in epidemiology were introduced in 1927 when Kermack and McKendrick published a series of papers that described the dynamics of disease transmission in terms of a system of differential equations [2].

Since the beginning of the widespread use of mathematical models for public health making, a large number of studies and publications have been made on modeling and analysis of epidemiological diseases. However, the majority of the studies were restricted to integer-order differential equations. For instance, the global stability of the SEIR and SEIAHR epidemic models with integer derivatives and different saturating contact rates were investigated in [3–6]. A delayed SIRS epidemic model with integer-order derivative involving saturation incidence and temporary immunity is studied in [7]. An epidemic stochastic mathematical model with integer order is used to predict the spread of coronavirus in [8]. A mathematical model is used to study correlation between the weather conditions and the COVID-19 pandemic in India by Borah et al. [9].

It has recently been turned out that fractional differential equations can successfully be used to model several phenomena in different fields including epidemiology [1]. Fractional calculus is a branch of mathematical analysis that studies calculus of derivatives and integrals of arbitrary orders.

The advantage of describing mathematical models using fractional derivatives is their nonlocal property in the sense that the *n*th derivative of a function $g(x)$ at *x* is a local property only when *n* is an integer, whereas a noninteger fractional derivative of $g(x)$ at $x=b$ depends on all values of $g(x)$ including those far away from *b*. In other words, in the case of fractional-order derivative, since it involves derivatives and integrals of arbitrary real or complex orders, the future state depends on the present and previous states. Using fractional-order derivatives in modeling, a dynamic system helps to describe the hereditary properties and efficacy (effectiveness, usefulness) of the memory as essential features in many biological mechanisms [10].

Many authors contributed to the development of fractional calculus starting from 1695 when L’Hospital asked Leibniz, what if the order of a derivative is $n=1/2$? Some of the other contributors include Euler in 1730 and Lagrange in 1849. In 1812, Laplace defined a fractional derivative using an integral, and in 1819 the derivative of arbitrary order appears in a text by Lacroix. Other prominent names in fractional derivatives and integrals include Reimann–Liouville, Hadamard, Caputo, Caputo–Fabrizio, and more recently Atangana–Baleanu. More historical notes and the nature of fractional calculus can be obtained in [11, 12] and the references therein.

In his recent publication, Atangana [13] exposed several previous mistakes and made corrections to the use of fractional calculus related to the fundamental theorem of calculus. Baleanu et al. [14] developed a fractional model for a tumor-immune surveillance mechanism and investigated the effect of chemotherapy treatment on the model. The result showed that the optimal control strategy was efficient. Sene [15] considered a fractional diffusion equation in the context of the fractional operator with Rabotnov fractional exponential kernel and determined the form of the analytical solution of the equation.

In [16] the Black–Scholes equation with Caputo–Fabrizio fractional derivative and a Mittag-Leffler fractional derivative is used to determine the value of an option, which plays an important role in finance. Analysis of a four-dimensional hyperchaotic system with Caputo–Liouville fractional derivative addressing the chaotic, hyperchaotic, and periodic behaviors of the system is considered in [17]. Kahan et al. [18] investigated a COVID-19 mathematical model with a fractal-fractional model in the sense of Atangana–Baleanu fractional operator. The issue of image processing with an Atangana–Baleanu fractional derivative in the sense of Caputo is pondered in [19]. New understandings in the existence of solution for Atangana–Baleanu Willis Aneurysm system and singular perturbation of boundary value problems for the nonlinear fuzzy differential equation are discussed by Panda et al. [20]. An insight on the existence and uniqueness of the solution to a COVID-19 mathematical model using fractional and fractional-fractal operators and fixed point theorem is performed in [21]. In [22, 23] a fractional-order mathematical model for COVID-19 is developed, and an investigation of the dynamics of the pandemic is performed. In [24] a fractal-fractional differentiation mathematical model is used for the analysis of diarrhea that occurred in Ghana during 2008–2018. Kahan et al. [10] analyzed HIV-TB coinfected mathematical model in the sense of Atangana–Baleanu fractional derivative.

Numerical computations of different ordinary and fractional derivatives were considered in [25–29]. Kolade and Owolabi [30] performed analysis and numerical simulation of a system using a two-step family of Adams–Bashforth method to approximate the Atangana–Baleanu fractional derivative. A SEIR fractional model with its stability analysis is considered in [31]. The Atangana–Baleanu fractional derivative operator involving the Mittag-Leffler kernel is used to analyze SEIRA mathematical model in [32].

From the above-surveyed works of the literature we can say that fractional derivatives have many applications in mathematical modeling and analysis of real phenomena. In particular, the recently developed Atangana–Baleanu fraction operator has earned popularity and respect due to its immense applications in biological, physical, medical engineering, and several other nonlinear analyses.

Motivated by the aforementioned arguments, in this paper, we study a SEAIR (Susceptible–Exposed–Asymptomatic–Symptomatic–Recovered cases) mathematical model involving a saturating contact rate. The recently developed Atangan–Baleanu fractional derivative and Toufic–Atangana numerical scheme [27] are used to develop the fractional derivative version of the model and estimate its numerical solution. To the best of the authors’ knowledge, the SEAIR endemic model applying the Atangana–Baleanu fractional derivative is not yet investigated. The authors also argue that optimal control analysis of mathematical models in the sense of Atangana–Baleanu fractional operators is uncommon in the existing literature. As a result, in this study, we consider an optimal control analysis of the SEAIR model. The rest of this paper is organized as follows. In Sect. 2, we accomplish the description and formulation of the model. In Sect. 3, we establish the existence and uniqueness of the solution of the model including the positivity and boundedness in the sense of the Atangana–Baleanu fractional operator. Section 4 deals with the diseases-free and endemic equilibrium points and the corresponding global stability analysis. The numerical solution of the SEAIR model via Atangana–Baleanu numerical scheme and simulation is detailed Sect. 5. In the last section, we consider an optimal control analysis of the fractional model by incorporating a control parameter in the model. Moreover, in this section, we perform a numerical simulation verifying the effect of the designed control strategy for different values of fractional order and different compartments of the model.

## Model description and formulation

In this section, we develop the Atangana–Baleanu fractional derivative representation of the SEAIR endemic mathematical model. Let us first recall the basic definitions of Atangana–Baleanu fractional operators.

### Definition 1

Let $g \in C^{1}(a,b)$, $a < b$, be a function, and let $\eta \in [0,1]$. The Atangana-Baleanu (AB) fractional derivative in Caputo type of order *η* is given by [25, 26, 33]

1 $$ {}_{a}^{{\mathrm{ABC}}}D_{t}^{\eta } g(t) = \frac{F(\eta )}{1 - \eta } \int _{a}^{t} \frac{{dg}}{{dk}} E_{\eta } \biggl[ - \frac{\eta }{1 - \eta } (t - k)^{\eta } \biggr] \, {dk}, $$

where $F(\eta )$ is the normalization function given by $F(\eta ) = 1 - \eta + \eta / \Gamma (\eta )$, characterized by $F(0) = F(1) = 1$, and the Mittag-Leffler function $E_{\eta } (z)$ with $\mathbb{C}$ the set of the complex number is given by

$$ E_{\eta } (z) = \sum_{\beta = 0}^{\infty } \frac{z^{\beta }}{\Gamma (1 + \eta \beta )},\quad \eta ,z \in \mathbb{C},\Re (\eta ) > 0. $$

### Definition 2

The AB fractional integral of the function $g \in C^{1}(a,b)$ is given by [25, 26, 33]

2 $$ {}_{a}^{{\mathrm{AB}}}I_{t}^{\eta } g(t) = \frac{1 - \eta }{F(\eta )}g(t) + \frac{\eta }{F(\eta ) \Gamma (\eta )} \int _{a}^{t} g(k) (t - k)^{\eta - 1} \, {dk}. $$

### Lemma 1

([34])

*The AB fractional derivative and AB fractional integral of the function* $g \in C^{1}(a,b)$ *satisfies the Newton–Leibniz equality*

$$ {}_{a}^{{\mathrm{AB}}}I_{t}^{\eta } \bigl({}_{a}^{{\mathrm{ABC}}}D_{t}^{\eta } g(t) \bigr) = g(t) - g(a). $$

### Lemma 2

([33, 35])

*For two functions* $f,g \in \Lambda ^{1}(a,b)$, $b > a$, *the AB fractional derivative satisfies the following inequality*:

$$ \bigl\Vert {}_{a}^{{\mathrm{ABC}}}D_{t}^{\eta } f(t) - {}_{a}^{{\mathrm{ABC}}}D_{t}^{\eta } g(t) \bigr\Vert \le \Lambda \bigl\Vert f(t) - g(t) \bigr\Vert . $$

**Figure 1:**
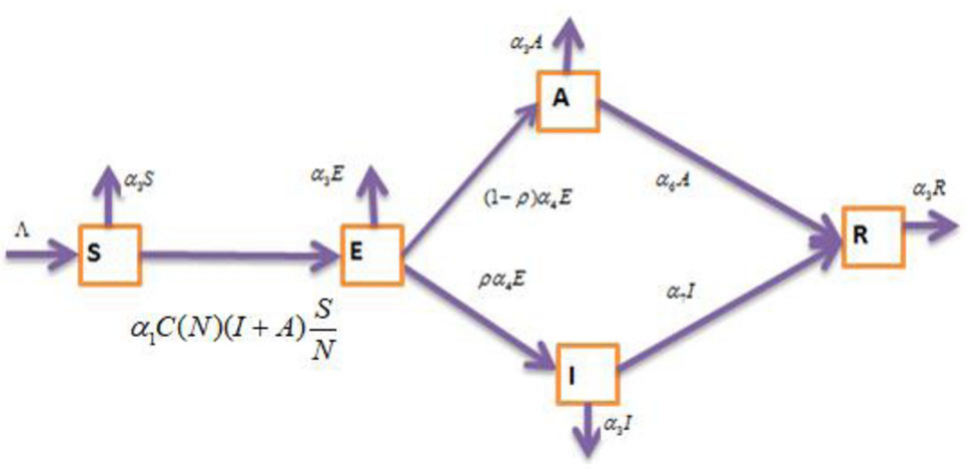
Flow diagram of SEAIR endemic model

Now we proceed with the formulation of the model. The following flow-diagram (Fig. 1) is used in constructing the mathematical model of this study.

Based on the flow diagram, the mathematical model with integer order used in this study is expressed by the equation system

3a $$ \left . \textstyle\begin{array}{l} \frac{{dS}}{{dt}} = \Lambda - \alpha _{1}C(N)(I + A)S - \alpha _{3}S, \\ \frac{{dE}}{{dt}} = \alpha _{1}C(N)(I + A)S - ( \alpha _{3} + \alpha _{4} )E, \\ \frac{{dA}}{{dt}} = (1 - \rho )\alpha _{4}E - ( \alpha _{3} + \alpha _{5} )A - \alpha _{6}A, \\ \frac{{dI}}{{dt}} = \rho \alpha _{4}E - ( \alpha _{3} + \alpha _{5} )I - \alpha _{7}I, \\ \frac{{dR}}{{dt}} = \alpha _{6}A + \alpha _{7}I - \alpha _{3}R, \end{array}\displaystyle \right \} $$

where $C(N) = \alpha N / (1 + {bN})$ derived in [36] is the saturating contact rate, *α* and *b* are positive constants, and the total population is given by $N(t) = S(t) + E(t) + A(t) + I(t) + R(t)$.

The natural death rate is proportional to the population size *N*; the death rate term is $\alpha _{3}N$. Thus in the absence of disease the differential equation of the total population size *N* is ${dN} / {dt} = \Lambda - \alpha _{3}N$, and thus $\lim_{x \to \infty } N(t) = \Lambda / \alpha _{3}$, which implies that the carrying capacity of the demographic structure under consideration in this study is $\Lambda / \alpha _{3}$. Moreover, we can see that $C(N) \approx \alpha N$ for small *N* and $C(N) \approx \alpha / b$ for large *N*; $C(N)$ is nondecreasing, and $C(N) / N$ is nonincreasing. We also consider the disease-induced death rate. We assume that once a patient is recovered, he/she develops a permanent immune, and there is no chance of returning to the susceptible group.

Thus the mathematical model taking into account the assumptions, the saturating contact rate, the flow diagram (Fig. 1), and the AB derivative is described by the system of differential equations

3b $$ \left . \textstyle\begin{array}{l} {}_{0}^{{\mathrm{ABC}}}D_{t}^{\eta } S(t) = G_{1}(t,S), \\ {}_{0}^{{\mathrm{ABC}}}D_{t}^{\eta } E(\tau ) = G_{2}(t,E), \\ {}_{0}^{{\mathrm{ABC}}}D_{t}^{\eta } A(t) = G_{3}(t,A), \\ {}_{0}^{{\mathrm{ABC}}}D_{t}^{\eta } I(t) = G_{4}(t,I), \\ {}_{0}^{{\mathrm{ABC}}}D_{t}^{\eta } R(t) = G_{5}(t,R), \end{array}\displaystyle \right \} $$

where the kernels are given by

$$ \left . \textstyle\begin{array}{l} G_{1}(t,S) = \Lambda - b_{0}\frac{(I + A)S}{k(N)} - \alpha _{3}S, \\ G_{2}(t,E) = b_{0}\frac{(I + A)S}{k(N)} - ( \alpha _{3} + \alpha _{4} )E, \\ G_{3}(t,A) = (1 - \rho )\alpha _{4}E - ( \alpha _{3} + \alpha _{5} + \alpha _{6} )A, \\ G_{4}(t,I) = \rho \alpha _{4}E - ( \alpha _{3} + \alpha _{5} + \alpha _{7} )I, \\ G_{5}(t,R) = \alpha _{6}A + \alpha _{7}I - \alpha _{3}R, \end{array}\displaystyle \right \} $$

with initial conditions $S(0) = S_{0}$, $E(0) = E_{0}$, $A(0) = A_{0}$, $I(0) = I_{0}$, $R(0) = R_{0}$, and $b_{0} = \alpha \alpha _{1}$, $k(N) = 1 + {bN}$, and $\alpha _{1}$ is the probability per unit time of transmitting the infection between two individuals taking part in contact.

In the presence of endemic, we have ${dN} / {dt} = \Lambda - \alpha _{3}N - \alpha _{5}(A + I)$, which indicates that the population size is not constant. The parameters used in the model are indicated in Table 1.

**Table 1:** The parameters and their descriptions

Parameter name	Symbol
Influx rate	Λ
Transmission rate from S to E due to contact with I and/or A	$\alpha _{1}$
Natural death rate	$\alpha _{3}$
The proportion of symptomatic infectious individuals	*ρ*
Rate of progression from E to A or I	$\alpha _{4}$
Disease induces death rate	$\alpha _{5}$
The recovery rate of asymptomatic cases	$\alpha _{6}$
The recovery rate of symptomatic cases	$\alpha _{7}$

## Existence and uniqueness of solutions

In this section, existence and uniqueness, nonnegativity, and boundedness of the solutions of the fractional-order model (3b) is deliberated. To show the existence of the solution to model (3b), we use the famous theorem referred to as the Banach fixed point theorem. For an extensive study on fixed points and contractions, we refer the reader to [21] and the references therein.

To show the existence and uniqueness of the solution, we proceed as follows. Applying the AB fractional integral to model (3b), we obtain

4 $$ \left . \textstyle\begin{array}{l} S(t) - S(0) = \frac{1 - \eta }{F(\eta )}G_{1}(t,S) + \frac{\eta }{F(\eta )\Gamma (\eta )}\int _{0}^{t} G_{1}(k,S) (t - k)^{\eta - 1}{\,dk}, \\ E(t) - E(0) = \frac{1 - \eta }{F(\eta )}G_{2}(t,E) + \frac{\eta }{F(\eta )\Gamma (\eta )}\int _{0}^{t} G_{2}(k,E) (t - k)^{\eta - 1}{\,dk}, \\ A(t) - A(0) = \frac{1 - \eta }{F(\eta )}G_{3}(t,A) + \frac{\eta }{F(\eta )\Gamma (\eta )}\int _{0}^{t} G_{3}(k,A) (t - k)^{\eta - 1}{\,dk}, \\ I(t) - I(0) = \frac{1 - \eta }{F(\eta )}G_{4}(t,I) + \frac{\eta }{F(\eta )\Gamma (\eta )}\int _{0}^{t} G_{4}(k,I) (t - k)^{\eta - 1}{\,dk}, \\ \mathrm{R}(t) - R(0) = \frac{1 - \eta }{F(\eta )}G_{5}(t,R) + \frac{\eta }{F(\eta )\Gamma (\eta )}\int _{0}^{t} G_{5}(k,R) (t - k)^{\eta - 1}{\,dk}. \end{array}\displaystyle \right \} $$

Consider the set $B = H(J) \times H(J) \times H(J) \times H(J) \times H(J)$ where $H(J) = C[0,T]$ is the Banach space of real-valued continuous functions defined on an interval $J = [0,T]$ with the corresponding norm defined by $\Vert ( S,E,A,I,R ) \Vert = \Vert S \Vert + \Vert E \Vert + \Vert A \Vert + \Vert I \Vert + \Vert R \Vert $, where

$$\begin{aligned}& \Vert S \Vert = \sup_{t \in J} \bigl\vert S(t) \bigr\vert , \qquad \Vert E \Vert = \sup_{t \in J} \bigl\vert E(t) \bigr\vert , \qquad \Vert A \Vert = \sup_{t \in J} \bigl\vert A(t) \bigr\vert , \\& \Vert I \Vert = \sup_{t \in J} \bigl\vert I(t) \bigr\vert , \qquad \Vert R \Vert = \sup_{t \in J} \bigl\vert R(t) \bigr\vert . \end{aligned}$$

### Theorem 1

(Lipschitz condition and contraction)

*For each of the kernels* $G_{1}$, $G_{2}$, $G_{3}$, $G_{4}$, $G_{5}$ *in* (3b), *there exists* $L_{i} > 0$, $i = 1,2,3,4,5$, *such that*

$$\begin{aligned}& \bigl\Vert G_{1}(t,S) - G_{1}(t,S_{1}) \bigr\Vert \le L_{1} \bigl\Vert S(t) - S_{1}(t) \bigr\Vert , \\& \bigl\Vert G_{2}(t,E) - G_{2}(t,E_{1}) \bigr\Vert \le L_{2} \bigl\Vert E(t) - E_{1}(t) \bigr\Vert , \\& \bigl\Vert G_{3}(t,A) - G_{3}(t,A_{1}) \bigr\Vert \le L_{3} \bigl\Vert A(t) - A_{1}(t) \bigr\Vert , \\& \bigl\Vert G_{4}(t,E) - G_{4}(t,E_{1}) \bigr\Vert \le L_{4} \bigl\Vert I(t) - I_{1}(t) \bigr\Vert , \\& \bigl\Vert G_{5}(t,R) - G_{5}(t,R_{1}) \bigr\Vert \le L_{5} \bigl\Vert R(t) - R_{1}(t) \bigr\Vert , \end{aligned}$$

*and are contractions for* $0 \le L_{i} < 1$, $i = 1,2,3,4,5$.

### Proof

$$\begin{aligned} \bigl\Vert G_{1}(t,S) - G_{1}(t,S_{1}) \bigr\Vert &= \biggl\Vert \Lambda - b_{0}\frac{(I + A)S}{k(N)} - \alpha _{3}S - \biggl(\Lambda - b_{0} \frac{(I + A)S_{1}}{k(N)} - \alpha _{3}S_{1}\biggr) \biggr\Vert \\ &= \biggl\Vert - b_{0}\frac{(I + A)S}{k(N)} - \alpha _{3}S + b_{0}\frac{(I + A)S_{1}}{k(N)} + \alpha _{3}S_{1} \biggr\Vert \\ &= \biggl\Vert b_{0}\frac{(I + A)}{k(N)} ( S_{1} - S ) + \alpha _{3} ( S_{1} - S ) \biggr\Vert \\ &\le \biggl( b_{0}\frac{m_{2} + m_{1}}{k(N)} + \alpha _{3} \biggr) \Vert S_{1} - S \Vert \\ &\le L_{1} \Vert S_{1} - S \Vert , \end{aligned}$$

where $L_{1} = b_{0}\frac{m_{2} + m_{1}}{k(N)} + \alpha _{3}$,

$$\begin{aligned}& \Vert S \Vert = \sup_{\tau \in J} \bigl\vert S(t) \bigr\vert = m_{5}, \qquad \Vert E \Vert = \sup_{\tau \in J} \bigl\vert E(t) \bigr\vert = m_{4}, \qquad \Vert A \Vert = \sup_{\tau \in J} \bigl\vert A(t) \bigr\vert = m_{2}, \\& \Vert I \Vert = \sup_{\tau \in J} \bigl\vert I(t) \bigr\vert = m_{1}, \qquad \Vert R \Vert = \sup_{\tau \in J} \bigl\vert R(t) \bigr\vert = m_{3}. \end{aligned}$$

It then follows that $G_{1}(t,S)$ satisfies the Lipschitz condition with Lipschitz constant $L_{1} = b_{0} ( m_{2} + m_{1} ) / k(N) + \alpha _{3}$. Moreover, if $0 \le L_{1} < 1$, then $G_{1}(t,S)$ is a contraction.

In the same manner, we can show the existence of $L_{i}$, $i = 2,3,4,5$, and a contraction principle for $G_{2}(t,E)$, $G_{3}(t,A)$, $G_{1}(t,I)$, $G_{1}(t,R) $, $0 \le L_{i} < 1$.

Now for $t = t_{n}$, $n = 1,2,\ldots $ , define the following recursive form of (4):

5 $$ \left . \textstyle\begin{array}{l} S_{n}(t) = \frac{1 - \eta }{F(\eta )}G_{1}(t,S_{n - 1}) + \frac{\eta }{F(\eta )\Gamma (\eta )}\int _{0}^{t} G_{1}(k,S_{n - 1})(t - k)^{\eta - 1}{\,dk}, \\ E_{n}(t) = \frac{1 - \eta }{F(\eta )}G_{2}(t,E_{n - 1}) + \frac{\eta }{F(\eta )\Gamma (\eta )}\int _{0}^{\tau } G_{2}(k,E_{n - 1})(t - k)^{\eta - 1}{\,dk}, \\ A_{n}(t) = \frac{1 - \eta }{F(\eta )}G_{3}(t,A_{n - 1}) + \frac{\eta }{F(\eta )\Gamma (\eta )}\int _{0}^{t} G_{3}(k,A_{n - 1})(t - k)^{\eta - 1}{\,dk}, \\ I_{n}(t) = \frac{1 - \eta }{F(\eta )}G_{4}(t,I_{n - 1}) + \frac{\eta }{F(\eta )\Gamma (\eta )}\int _{0}^{t} G_{4}(k,I_{n - 1})(t - k)^{\eta - 1}{\,dk}, \\ R_{n}(t) = \frac{1 - \eta }{F(\eta )}G_{5}(t,R_{n - 1}) + \frac{\eta }{F(\eta )\Gamma (\eta )}\int _{0}^{t} G_{5}(k,R_{n - 1})(t - k)^{\eta - 1}{\,dk}, \end{array}\displaystyle \right \} $$

with initial conditions $S_{0}(t) = S(0)$, $E_{0}(t) = E(0)$, $A_{0}(t) = A(0)$, $I_{0}(t) = I(0)$, $R_{0}(t) = R(0)$.

The differences between successive terms in (5) are expressed as follows:

6 $$ \left . \textstyle\begin{array}{l} A_{1n}(t) = S_{n}(t) - S_{n - 1}(t) \\ \hphantom{A_{1n}(t)}= \frac{1 - \eta }{F(\eta )} ( G_{1}(t,S_{n - 1}) - G_{1}(t,S_{n - 2}) ) \\ \hphantom{A_{1n}(t)={}}{}+ \frac{\eta }{F(\eta )\Gamma (\eta )}\int _{0}^{t} ( G_{1}(t,S_{n - 1}) - G_{1}(t,S_{n - 2}) )(t - k)^{\eta - 1}{\,dk}, \\ A_{2n}(t) = E_{n}(t) - E_{n - 1}(t) \\ \hphantom{A_{2n}(t)}= \frac{1 - \eta }{F(\eta )} ( G_{2}(t,E_{n - 1}) - G_{2}(t,E_{n - 2}) ) \\ \hphantom{A_{2n}(t)={}}{}+ \frac{\eta }{F(\eta )\Gamma (\eta )}\int _{0}^{t} ( G_{1}(t,E_{n - 1}) - G_{1}(t,E_{n - 2}) )(t - k)^{\eta - 1}{\,dk}, \\ A_{3n}(t) = A_{n}(t) - A_{n - 1}(t) \\ \hphantom{A_{3n}(t)}= \frac{1 - \eta }{F(\eta )} ( G_{3}(t,A_{n - 1}) - G_{3}(t,A_{n - 2}) ) \\ \hphantom{A_{3n}(t)={}}{}+ \frac{\eta }{F(\eta )\Gamma (\eta )}\int _{0}^{t} ( G_{3}(t,A_{n - 1}) - G_{3}(t,A_{n - 2}) )(t - k)^{\eta - 1}{\,dk}, \\ A_{4n}(t) = I_{n}(t) - I_{n - 1}(t) \\ \hphantom{A_{4n}(t)}= \frac{1 - \eta }{F(\eta )} ( G_{4}(t,I_{n - 1}) - G_{4}(t,I_{n - 2}) ) \\ \hphantom{A_{4n}(t)={}}{}+ \frac{\eta }{F(\eta )\Gamma (\eta )}\int _{0}^{t} ( G_{1}(t,I_{n - 1}) - G_{1}(t,I_{n - 2}) )(t - k)^{\eta - 1}{\,dk}, \\ A_{5n}(t) = R_{n}(t) - R_{n - 1}(t) \\ \hphantom{A_{5n}(t)}= \frac{1 - \eta }{F(\eta )} ( G_{5}(t,R_{n - 1}) - G_{5}(t,R_{n - 2}) ) \\ \hphantom{A_{5n}(t)={}}{}+ \frac{\eta }{F(\eta )\Gamma (\eta )}\int _{0}^{t} ( G_{5}(t,R_{n - 1}) - G_{5}(t,R_{n - 2}) )(t - k)^{\eta - 1}{\,dk}. \end{array}\displaystyle \right \} $$

Taking the norm on both sides of each equation in (6), we have

7 $$ \left . \textstyle\begin{array}{l} \Vert A_{1n}(t) \Vert = \Vert S_{n}(t) - S_{n - 1}(t) \Vert \\ \hphantom{\Vert A_{1n}(t) \Vert }= \frac{1 - \eta }{F(\eta )} \Vert G_{1}(t,S_{n - 1}) - G_{1}(t,S_{n - 2}) \Vert \\ \hphantom{\Vert A_{1n}(t) \Vert ={}}{}+ \frac{\eta }{F(\eta )\Gamma (\eta )}\int _{0}^{t} \Vert G_{1}(t,S_{n - 1}) - G_{1}(t,S_{n - 2}) \Vert (t - k)^{\eta - 1}{\,dk}, \\ \Vert A_{2n}(t) \Vert = \Vert E_{n}(t) - E_{n - 1}(t) \Vert \\ \hphantom{\Vert A_{2n}(t) \Vert }= \frac{1 - \eta }{F(\eta )} \Vert G_{2}(t,E_{n - 1}) - G_{2}(t,E_{n - 2}) \Vert \\ \hphantom{\Vert A_{2n}(t) \Vert ={}}{}+ \frac{\eta }{F(\eta )\Gamma (\eta )}\int _{0}^{t} \Vert G_{1}(t,E_{n - 1}) - G_{1}(t,E_{n - 2}) \Vert (t - k)^{\eta - 1}{\,dk}, \\ \Vert A_{3n}(t) \Vert = \Vert A_{n}(t) - A_{n - 1}(t) \Vert \\ \hphantom{\Vert A_{3n}(t) \Vert }= \frac{1 - \eta }{F(\eta )} \Vert G_{3}(t,A_{n - 1}) - G_{3}(t,A_{n - 2}) \Vert \\ \hphantom{\Vert A_{3n}(t) \Vert ={}}{}+ \frac{\eta }{F(\eta )\Gamma (\eta )}\int _{0}^{t} \Vert G_{3}(t,A_{n - 1}) - G_{3}(t,A_{n - 2}) \Vert (t - k)^{\eta - 1}{\,dk}, \\ \Vert A_{4n}(t) \Vert = \Vert I_{n}(t) - I_{n - 1}(t) \Vert \\ \hphantom{\Vert A_{4n}(t) \Vert }= \frac{1 - \eta }{F(\eta )} \Vert G_{4}(t,I_{n - 1}) - G_{4}(t,I_{n - 2}) \Vert \\ \hphantom{\Vert A_{4n}(t) \Vert ={}}{}+ \frac{\eta }{F(\eta )\Gamma (\eta )}\int _{0}^{t} \Vert G_{1}(t,I_{n - 1}) - G_{1}(t,I_{n - 2}) \Vert (t - k)^{\eta - 1}{\,dk}, \\ \Vert A_{5n}(t) \Vert = \Vert R_{n}(t) - R_{n - 1}(t) \Vert \\ \hphantom{\Vert A_{5n}(t) \Vert }= \frac{1 - \eta }{F(\eta )} \Vert G_{5}(t,R_{n - 1}) - G_{5}(t,R_{n - 2}) \Vert \\ \hphantom{\Vert A_{5n}(t) \Vert ={}}{}+ \frac{\eta }{F(\eta )\Gamma (\eta )}\int _{0}^{t} \Vert G_{5}(t,R_{n - 1}) - G_{5}(t,R_{n - 2}) \Vert (t - k)^{\eta - 1}{\,dk}. \end{array}\displaystyle \right \} $$

Furthermore, the first equality in (7) can be reduced to the following expressions:

$$\begin{aligned} \bigl\Vert A_{1n}(t) \bigr\Vert =& \bigl\Vert S_{n}(t) - S_{n - 1}(t) \bigr\Vert \\ \le& \frac{1 - \eta }{F(\eta )} \bigl\Vert \bigl( G_{1}(t,S_{n - 1}) - G_{1}(t,S_{n - 2}) \bigr) \bigr\Vert \\ &{}+\frac{\eta }{F(\eta )\Gamma (\eta )} \int _{0}^{\tau } \bigl\Vert \bigl( G_{1}(t,S_{n - 1}) - G_{1}(t,S_{n - 2}) \bigr) \bigr\Vert (t - k)^{\eta - 1}{\,dk} \\ \le& \frac{1 - \eta }{F(\eta )}L_{1} \Vert S_{n - 1} - S_{n - 2} \Vert + \frac{\eta }{F(\eta )\Gamma (\eta )}L_{1} \int _{0}^{\tau } \Vert S_{n - 1} - S_{n - 2} \Vert (t - k)^{\eta - 1}{\,dk} \\ \le& L_{1} \bigl\Vert A_{1 ( n - 1 )}(t) \bigr\Vert \biggl\vert \frac{1 - \eta }{F(\eta )} + \frac{t^{\eta }}{F(\eta )\Gamma (\eta )} \biggr\vert . \end{aligned}$$

As a result, we have

8 $$ \bigl\Vert A_{1n}(t) \bigr\Vert \le L_{1} \biggl\vert \frac{1 - \eta }{F(\eta )} + \frac{t^{\eta }}{F(\eta )\Gamma (\eta )} \biggr\vert \bigl\Vert A_{1 ( n - 1 )}(t) \bigr\Vert . $$

Analogously, the remaining expressions of (7) can be reduced to the following expressions:

9 $$ \left . \textstyle\begin{array}{l} \Vert A_{2n}(t) \Vert \le L_{2} \vert \frac{1 - \eta }{F(\eta )} + \frac{t^{\eta }}{F(\eta )\Gamma (\eta )} \vert \Vert A_{2 ( n - 1 )}(t) \Vert , \\ \Vert A_{3n}(t) \Vert \le L_{3} \vert \frac{1 - \eta }{F(\eta )} + \frac{t^{\eta }}{F(\eta )\Gamma (\eta )} \vert \Vert A_{3 ( n - 1 )}(t) \Vert , \\ \Vert A_{4n}(t) \Vert \le L_{4} \vert \frac{1 - \eta }{F(\eta )} + \frac{t^{\eta }}{F(\eta )\Gamma (\eta )} \vert \Vert A_{4 ( n - 1 )}(t) \Vert , \\ \Vert A_{5n}(t) \Vert \le L_{5} \vert \frac{1 - \eta }{F(\eta )} + \frac{t^{\eta }}{F(\eta )\Gamma (\eta )} \vert \Vert A_{5 ( n - 1 )}(t) \Vert . \end{array}\displaystyle \right \} $$

 □

### Theorem 2

*The AB fractional model given in* (3b) *has a solution if we can find* $M_{0}$ *satisfying the inequality*

10 $$ \biggl( \frac{1 - \eta }{F(\eta )} + \frac{M_{{0}}^{\eta }}{F(\eta )\Gamma (\eta )} \biggr)L_{i} < 1,\quad i = 1,2,3,4,5. $$

### Proof

From (8) and (9) we have

11 $$ \left . \textstyle\begin{array}{l} \Vert A_{1n}(t) \Vert \le \Vert S(0) \Vert [ ( \frac{1 - \eta }{F(\eta )} + \frac{M_{0}^{\eta }}{F(\eta )\Gamma (\eta )} )L_{1} ]^{n}, \\ \Vert A_{2n}(t) \Vert \le \Vert E(0) \Vert [ ( \frac{1 - \eta }{F(\eta )} + \frac{M_{0}^{\eta }}{F(\eta )\Gamma (\eta )} )L_{2} ]^{n}, \\ \Vert A_{3n}(t) \Vert \le \Vert A(0) \Vert [ ( \frac{1 - \eta }{F(\eta )} + \frac{M_{0}^{\eta }}{F(\eta )\Gamma (\eta )} )L_{3} ]^{n}, \\ \Vert A_{4n}(t) \Vert \le \Vert I(0) \Vert [ ( \frac{1 - \eta }{F(\eta )} + \frac{M_{0}^{\eta }}{F(\eta )\Gamma (\eta )} )L_{4} ]^{n}, \\ \Vert A_{5n}(t) \Vert \le \Vert R(0) \Vert [ ( \frac{1 - \eta }{F(\eta )} + \frac{M_{0}^{\eta }}{F(\eta )\Gamma (\eta )} )L_{5} ]^{n}. \end{array}\displaystyle \right \} $$

The existence of the solution (the existence of a fixed point) is confirmed by Theorem 1, and we have to show that the functions $S(t)$, $E(t)$, $A(t)$, $I(t)$, $R(t)$ are solutions of model (3b).

Let us assume that the following are satisfied:

12 $$ \left . \textstyle\begin{array}{l} S(t) - S(0) = S_{n}(t) - a_{1n}(t), \\ E(t) - E(0) = E_{n}(t) - a_{2n}(t), \\ A(t) - A(0) = A_{n}(t) - a_{3n}(t), \\ I(t) - I(0) = I_{n}(t) - a_{4n}(t), \\ R(t) - R(0) = R_{n}(t) - a_{5n}(t). \end{array}\displaystyle \right \} $$

From (12) we obtain

$$\begin{aligned} \bigl\Vert a_{1n}(t) \bigr\Vert \le& \frac{1 - \eta }{F(\eta )} \bigl\Vert \bigl( G_{1}(\tau ,S_{n}) - G_{1}(\tau ,S_{n - 1}) \bigr) \bigr\Vert \\ &{}+ \frac{\eta }{F(\eta )\Gamma (\eta )} \int _{0}^{\tau } \bigl\Vert \bigl( G_{1}(\tau ,S_{n}) - G_{1}(\tau ,S_{n - 1}) \bigr) \bigr\Vert (\tau - k)^{\eta - 1}{\,dk}, \\ \le& \frac{1 - \eta }{F(\eta )}L_{1} \Vert S_{n} - S_{n - 1} \Vert + \frac{\eta ^{n}}{F(\eta )\Gamma (\eta )}L_{1} \Vert S_{n} - S_{n - 1} \Vert . \end{aligned}$$

Repeating the process recursively leads to

$$ \bigl\Vert a_{1n}(t) \bigr\Vert \le \biggl[ \frac{1 - \eta }{F(\eta )} + \frac{t^{\eta }}{F(\eta )\Gamma (\eta )} \biggr]^{n + 1}L_{1}^{n} \Vert S_{n} - S_{n - 1} \Vert ^{n}, $$

which at $t = M_{{0}}^{\eta } $ yields

13 $$ \begin{aligned} &\bigl\Vert a_{1n}(t) \bigr\Vert \le \biggl[ \frac{1 - \eta }{F(\eta )} + \frac{M_{o}^{\eta }}{F(\eta )\Gamma (\eta )} \biggr]^{n + 1}L_{1}^{n} \Vert S_{n} - S_{n - 1} \Vert ^{n}, \\ & \bigl\Vert a_{1n}(t) \bigr\Vert \to 0. \end{aligned} $$

Applying the limit to both sides of (13) as $n \to \infty $, we see that $\Vert a_{1n}(t) \Vert \to 0$ for

$$ \biggl( \frac{1 - \eta }{F(\eta )} + \frac{t^{\eta }}{F(\eta )\Gamma (\eta )} \biggr)L_{1} < 1 . $$

Similarly, we can show that $\Vert a_{2n}(t) \Vert \to 0$, $\Vert a_{3n}(t) \Vert \to 0$, $\Vert a_{4n}(t) \Vert \to 0$, $\Vert a_{5n}(t) \Vert \to 0$,

$$ \biggl( \frac{1 - \eta }{F(\eta )} + \frac{t^{\eta }}{F(\eta )\Gamma (\eta )} \biggr)L_{i} < 1, \quad i = 2,3,4,5 . $$

 □

Theorems 1 and 2 guarantee the existence of the solution of model (3b) by the Banach fixed point theorem. The uniqueness of the solution is proved in Theorem 3.

### Theorem 3

(Uniqueness of solution)

*The AB fractional model* (3b) *has a unique solution*, *provided that*

14 $$ \biggl( \frac{1 - \eta }{F(\eta )} + \frac{t^{\eta }}{F(\eta )\Gamma (\eta )} \biggr)L_{i} < 1,\quad i = 2,3,4,5. $$

### Proof

Let us assume that $S_{1}(t)$, $E_{1}(t)$, $A_{1}(t)$, $I_{1}(t)$, $R_{1}(t)$ are also solutions to (3b). Then

$$ S(t) - S_{1}(t) = \frac{1 - \eta }{F(\eta )} \bigl( G_{1}(t,S) - G_{1}(t,S_{1}) \bigr) + \frac{\eta }{F(\eta )\Gamma (\eta )} \int _{0}^{t} \bigl( G_{1}(t,S) - G_{1}(t,S_{1}) \bigr) (t - k)^{\eta - 1}{\,dk}. $$

Taking the norm of both sides, we obtain

$$ \bigl\Vert S(t) - S_{1}(t) \bigr\Vert \le \frac{1 - \eta }{F(\eta )}L_{1} \Vert S - S_{1} \Vert + \frac{t^{\eta }}{F(\eta )\Gamma (\eta )}L_{1} \Vert S - S_{1} \Vert . $$

Since $( 1 - L_{1} ( \frac{1 - \eta }{F(\eta )} + \frac{t^{\eta }}{F(\eta )\Gamma (\eta )} ) ) > 0$, we obtain $\Vert S(t) - S_{1}(t) \Vert = 0 $. Thus we have $S(t) = S_{1}(t)$.

Similarly, we can show that $E(t) = E_{1}(t)$, $A(t) = A_{1}(t)$, $I(t) = I_{1}(t)$, $R(t) = R_{1}(t)$, which completes the proof of Theorem 3. □

Now we define the epidemiologically feasible (nonnegativity and boundedness) region of this study in Theorem 4 and prove that the region is positively invariant and bounded.

### Theorem 4

*The epidemiologically feasible region of AB model* (3b) *is given by*

15 $$ \Omega =: \biggl\{ ( S,E,A,I,R ) \in R_{ +}^{5}:0 \le S + E + A + I + R \le N \le \frac{\Lambda }{\alpha _{3}} \biggr\} . $$

The existence and uniqueness of the solution of model (3b) are now proved, and it remains to show that the set Ω defined in (15) is positively invariant. The following lemma will be used for the proof of Theorem 4.

### Lemma 3

(Generalized mean value theorem, [37])

*Let* $g(x) \in C[a,b]$, *and let* ${}_{0}^{{\mathrm{ABC}}}D_{t}^{\eta } g(x) \in C[a,b]$ *when* $0 < \eta \le 1$. *Then we have* $g(x) = g(a) + \frac{1}{\Gamma (\eta )}{}_{0}^{{\mathrm{ABC}}}D_{t}^{\eta } g(\xi )(x - a)^{\eta }$,*when* $0 \le \xi \le x$, $\forall x \in (a,b]$.

Note that by Lemma 3, if $g(x) \in [0,b]$, ${}_{0}^{{\mathrm{ABC}}}D_{t}^{\eta } g(x) \in (0,b]$, and ${}_{0}^{{\mathrm{ABC}}}D_{t}^{\eta } g(x) \ge 0$, $\forall x \in (0,b]$ when $0 < \eta \le 1$, then the function $g(x)$ is nondecreasing, and if ${}_{0}^{{\mathrm{ABC}}}D_{t}^{\eta } g(x) \le 0$, $\forall x \in (0,b]$, then the function $g(x)$ is nonincreasing $\forall x \in [0,b]$.

To show that the set Ω is positively invariant, using Lemma 3, we have

16 $$ \left . \textstyle\begin{array}{l} {}_{0}^{{\mathrm{ABC}}}D_{t}^{\eta } S|_{S = 0} = \Lambda \ge 0, \\ D_{t}^{\eta } E|_{E = 0} = b_{0}\frac{(I + A)S}{k(N)} \ge 0, \\ {}_{0}^{{\mathrm{ABC}}}D_{t}^{\eta } A|_{A = 0} = (1 - \rho )\alpha _{4}E \ge 0, \\ {}_{0}^{{\mathrm{ABC}}}D_{t}^{\eta } I|_{I = 0} = \rho \alpha _{4}E \ge 0, \\ {}_{0}^{{\mathrm{ABC}}}D_{t}^{\eta } R|_{R = 0} = \alpha _{6}A + \alpha _{7}I \ge 0. \end{array}\displaystyle \right \} $$

It follows from (16) that each of the solutions of (3b) is nonnegative and remains in $R_{ +}^{5}$, and hence the set Ω defined in (15) is positively invariant for model (3b).

Finally, to establish the boundedness of the solutions of the fractional model (3b), taking into account that all the parameters are positive, we continue by summing all the equations of the model, which gives

$$ {}_{0}^{{\mathrm{ABC}}}D_{t}^{\eta } N(t) = \Lambda - \alpha _{3}N(t) - \alpha _{5}(A + I) \le \Lambda - \alpha _{3}N(t). $$

Applying the Laplace transform leads to

$$\begin{aligned}& L \bigl( {}_{0}^{{\mathrm{ABC}}}D_{t}^{\eta } N(t) + \alpha _{3}N(t) \bigr) \le L ( \Lambda ), \\& L(N) \biggl( (1 - k)s^{\eta } - \frac{k\eta }{1 - \eta } \biggr) - s^{\eta - 1}N(0) \le \frac{1 - \eta }{F(\eta )} \biggl( s^{\eta } + \frac{\eta }{1 - \eta } \biggr)\frac{\Lambda }{s}, \\& L(N) \le \biggl( 1 - \frac{k\eta }{(1 - k)(1 - \eta )}s^{ - \eta } \biggr)^{ - 1} \biggl[ \frac{1 - \eta }{ ( 1 - k )F(\eta )} \biggl( 1 + \frac{\eta }{1 - \eta } s^{ - \eta } \biggr)\frac{\Lambda }{s} + N(0) \frac{1}{ ( 1 - k )s} \biggr], \end{aligned}$$

where

$$ k = \frac{ - \alpha _{3}(1 - \eta )}{F(\eta )}. $$

Following the work [38] and applying the inverse Laplace transform, the solution is given by

$$\begin{aligned} N(t) =& \frac{\Lambda }{\alpha _{3}} - \frac{\Lambda }{\alpha _{3}(1 - k)}\frac{d}{{dt}} \int _{0}^{t} E_{\eta } \biggl( \frac{k\eta }{(1 - k)(1 - \eta )}(t - x)^{\eta } {\,dx} \biggr) \\ &{}+ \frac{1}{1 - k}E_{\eta } \biggl( \frac{k\eta }{(1 - k)(1 - \eta )}t^{\eta } \biggr)N(0), \end{aligned}$$

where $E_{\alpha ,\beta } $ refers to the Mittag-Leffler function. Taking into account the fact that the Mittag-Leffler function has the asymptotic behavior

$$ E_{\alpha ,\beta } (z) \approx \sum_{K = 1}^{\omega } z^{ - K} / \Gamma (\beta - \alpha K) + \mathrm{O} \bigl( \vert z \vert ^{ - 1 - \omega } \bigr),\quad \vert z \vert \to \infty , \frac{\alpha \pi }{2} < \bigl\vert \arg (z) \bigr\vert \le \pi , $$

it is not difficult to observe that $N(t) \to \Lambda / \alpha _{3}$ as $t \to \infty $. Hence (15) is the biologically feasible region of model (3b).

## Equilibrium points and stability analysis

I. *The disease-free equilibrium point (DFE)*

The disease-free equilibrium of (3b) is given by $N_{0} = (\Lambda / \alpha _{3},0,0,0,0) \in \partial \Omega $. The global stability of the disease-free equilibrium point is proved in Theorem 5 after defining the basic reproductive number.

II. *The basic reproductive number* $R_{0}$

The basic reproductive number is obtained using the next-generation matrix [39] and is the spectral radius $\Phi ( - {TV}^{ - 1})$, where

$$\begin{aligned}& T = \begin{bmatrix} 0 & \frac{b_{0}\Lambda }{\alpha _{3}k(N)} & \frac{b_{0}\Lambda }{\alpha _{3}k(N)} \\ 0 & 0 & 0 \\ 0 & 0 & 0 \end{bmatrix},\qquad - V^{ - 1} = \begin{bmatrix} \frac{1}{\alpha _{4} + \alpha _{3}} & 0 & 0 \\ \frac{\alpha _{4}(1 - \rho )}{l_{1}(\alpha _{4} + \alpha _{3})} & \frac{1}{l_{1}} & 0 \\ \frac{\alpha _{4}\rho }{l_{2}(\alpha _{4} + \alpha _{3})} & 0 & \frac{1}{l_{2}} \end{bmatrix}, \\& \Phi \bigl( - {TV}^{ - 1} \bigr) = \frac{(1 - \rho )\Lambda b_{0}\alpha _{4}l_{2} + \rho \Lambda b_{0}\alpha _{4}l_{1}}{l_{1}l_{2} ( \alpha _{3} + b\Lambda )(\alpha _{3} + \alpha _{4})} = R_{0} = R_{0I} + R_{0A}, \\& R_{0I} = \frac{\rho \Lambda b_{0}\alpha _{4}}{ ( b\Lambda + \alpha _{3} )l_{2}(\alpha _{3} + \alpha _{4})}, \qquad R_{0A} = \frac{(1 - \rho )\Lambda b_{0}\alpha _{4}}{l_{1} ( \alpha _{3} + b\Lambda )(\alpha _{3} + \alpha _{4})}, \\& l_{1} = \alpha _{3} + \alpha _{5} + \alpha _{6}, \qquad l_{2} = \alpha _{3} + \alpha _{5} + \alpha _{7}. \end{aligned}$$

### Theorem 5

*The DFE*; $N_{0} = (\Lambda / \alpha _{3},0,0,0,0)$ *is globally asymptotically stable when* $R_{0} \le 1$.

### Proof

Consider a Lyapunov function candidate

$$ V(S,E,A,I,R) = \alpha _{4}E + ( \alpha _{3} + \alpha _{4} ) \bigl((1 - \rho )A + \rho I \bigr). $$

The derivative of *V* in the direction of the solution of (3b) is given as

$$\begin{aligned} \frac{{dV}}{{dt}} =& \alpha _{4}\frac{{dE}}{{dt}} + ( \alpha _{3} + \alpha _{4} ) \biggl( (1 - \rho ) \frac{{dA}}{{dt}} + \rho \frac{{dI}}{{dt}} \biggr) \\ =& \biggl( b_{0}\alpha _{4}\frac{A ( N - E - A - I - R )}{k(N)} \biggr) + \biggl( b_{0}\alpha _{4}\frac{I ( N - E - A - I - R )}{k(N)} \biggr) \\ &{}- \bigl( ( \alpha _{3} + \alpha _{4} + \alpha _{6} ) ( \alpha _{3} + \alpha _{4} )A + ( \alpha _{3} + \alpha _{4} + \alpha _{7} ) ( \alpha _{3} + \alpha _{4} )I \bigr) \\ =& b_{0}\alpha _{4}(A + I)\frac{N}{k(N)} - \frac{b_{0}\alpha _{4}A(E + A + I + R)}{k(N)} - \frac{b_{0}\alpha _{4}I(E + A + I + R)}{k(N)}. \\ &{}- \bigl( ( \alpha _{3} + \alpha _{4} + \alpha _{6} ) ( \alpha _{3} + \alpha _{4} )A + ( \alpha _{3} + \alpha _{4} + \alpha _{7} ) ( \alpha _{3} + \alpha _{4} )I \bigr) \\ \le& b_{0}\alpha _{4}(A + I)\frac{N}{k(N)} - \bigl( ( \alpha _{3} + \alpha _{4} + \alpha _{6} ) ( \alpha _{3} + \alpha _{4} )A + ( \alpha _{3} + \alpha _{4} + \alpha _{7} ) ( \alpha _{3} + \alpha _{4} )I \bigr) \\ \le& b_{0}\alpha _{4}A\frac{N}{k(N)} - \bigl( ( \alpha _{3} + \alpha _{4} + \alpha _{6} ) ( \alpha _{3} + \alpha _{4} )A \bigr) + b_{0}\alpha _{4}(I)\frac{N}{k(N)} \\ &{}- \bigl( ( \alpha _{3} + \alpha _{4} + \alpha _{7} ) ( \alpha _{3} + \alpha _{4} )I \bigr) \\ \le& ( \alpha _{3} + \alpha _{4} + \alpha _{6} ) ( \alpha _{3} + \alpha _{4} )A \biggl( R_{0A}\frac{C(N)}{C(\Lambda / \alpha _{3})} - 1 \biggr) \\ &{}+ ( \alpha _{3} + \alpha _{4} + \alpha _{7} ) ( \alpha _{3} + \alpha _{4} )I \biggl( R_{0I}\frac{C(N)}{C(\Lambda / \alpha _{3})} - 1 \biggr). \end{aligned}$$

Thus

$$\begin{aligned} \frac{{dV}}{d\tau } \le& ( \alpha _{3} + \alpha _{4} + \alpha _{6} ) ( \alpha _{3} + \alpha _{4} )A \biggl( R_{0A}\frac{C(N)}{C(\Lambda / \alpha _{3})} - 1 \biggr) \\ &{}+ ( \alpha _{3} + \alpha _{4} + \alpha _{7} ) ( \alpha _{3} + \alpha _{4} )I \biggl( R_{0I}\frac{C(N)}{C(\Lambda / \alpha _{3})} - 1 \biggr) \le 0, \end{aligned}$$

for $R_{0A} \le 1$, $R_{0I} \le 1$ and $R_{0} = R_{0A} + R_{0I} \le 1$.

Since in the set Ω we have $N = \Lambda / \alpha _{3}$ at the DFE and $C(N) = \alpha N / (1 + {bN})$ is nondecreasing, $\frac{{dV}}{{dt}} \le 0$ for $R_{0A} \le 1$, $R_{0I} \le 1$ and $R_{0} = R_{0A} + R_{0I} \le 1$. Moreover, the Lyapunov–Lasalle theorem [40] implies that all trajectories in Ω approach the largest positively invariant subset of the set M where ${dV} / {dt} = 0$. In this work, M is the set where $I = A = 0$. On the boundary of Ω, where $I = A = 0$, we have $E = 0$, $R(t) = c_{0}e^{ - t} \to 0$ as $t \to + \infty $, and $N(t) = \Lambda / \alpha _{3} + (N(0) - \Lambda / \alpha _{3})e^{ - t} \to \Lambda / \alpha _{3}$ as $t \to + \infty $. □

Hence all the trajectories in the domain Ω approach the disease-free equilibrium point $N_{0} = (\Lambda / \alpha _{3},0,0,0,0)$, for $R_{0} \le 1$.

III. *Existence and uniqueness of endemic equilibrium point (EEP)*

The proof of the global stability of the DFE ensures that when $R_{0} \le 1$, there is no other equilibrium point other than $N_{0} = (\Lambda / \alpha _{3},0,0,0,0)$. As a result, the study of the endemic equilibrium point $N^{*} = (S^{*},E^{*},A^{*},I^{*},R^{*})$ is restricted to $R_{0} > 1$.

The endemic equilibrium point is given by $N^{*} = (S^{*},E^{*},A^{*},I^{*},R^{*})$, where

17 $$ \begin{aligned} &A^{*} = \frac{(1 - \rho )\alpha _{{4}}}{l_{1}}E^{*}, \qquad I^{*} = \frac{\rho \alpha _{{4}}}{l_{2}}E^{*},\qquad R^{*} = \biggl( \frac{ ( 1 - \rho )\alpha _{{4}}\alpha _{{6}}}{\alpha _{3}l_{1}} + \frac{\rho \alpha _{{7}}\alpha _{{4}}}{\alpha _{3}l_{2}} \biggr)E^{*}, \\ &S^{*} = \frac{\Lambda }{\alpha _{3}} - \biggl( \frac{\alpha _{3} + \alpha _{4}}{\alpha _{3}} \biggr)E^{*}, \qquad E^{*} = (R_{0} - \alpha _{3}) / \bigl((\alpha _{3} + \alpha _{4})R_{0}\bigr), \end{aligned} $$

and $E^{*}$ satisfies the quadratic equation $( \frac{R_{0}(\alpha _{3} + \alpha _{4})}{\alpha _{3}} - (\alpha _{3} + \alpha _{4}) )E^{*} - \frac{R_{0}(\alpha _{3} + \alpha _{4})^{2}}{\alpha _{3}}E^{* 2} = 0$, from which we have that $E^{*}$ is positive only for $R_{0} > \max \{ 1, \alpha _{3} \} $. It is logical to assume that the natural death rate $\alpha _{3} \le 1$. Hence we conclude that the EEP is globally asymptotically stable for $R_{0} > 1$.

To show the uniqueness of EEP, with the assumption of $E^{*} > 0$, define

$$ G \bigl(E^{*} \bigr) =: \biggl( \frac{R_{0}}{\alpha _{3}} - 1 \biggr) - \frac{R_{0}(\alpha _{3} + \alpha _{4})}{\alpha _{3}}E^{*}, $$

and then we have ${dG} / {dE}^{*} = - R_{0}(\alpha _{3} + \alpha _{4}) / \alpha _{3} < 0$, that is, $G(E^{*})$ is a decreasing function in $(0, \Lambda/\alpha_{3})\in \Omega$, ensuring that $E^{*} \in ( 0,\Lambda / \alpha _{3} )$ is unique, which leads to Theorem 6.

### Theorem 6

*For* $R_{0} > 1$, *model* (3b) *has a unique equilibrium point* $N^{*} = (E^{*},A^{*},I^{*},R^{*},S^{*})$ *given by* (17). *The global stability of the EEP is proved in Theorem *7*using the Lyapunov function method*.

### Theorem 7

*If* $R_{0} > 1$, *then the endemic equilibrium point* $N^{*}$ *of model* (3b) *is globally asymptotically stable in the region* Ω.

### Proof

Define a Lyapunov function candidate by

$$ F(S,E,I,A,R) = \frac{1}{2} \bigl[ \bigl(S - S^{*} \bigr) + \bigl(E - E^{*} \bigr) + \bigl(I - I^{*} \bigr) + \bigl(A - A^{*} \bigr) + \bigl(R - R^{*} \bigr) \bigr]^{2}. $$

Then $F(S,E,I,A,R) \ge 0$ and $F(S^{*},E^{*},I^{*},A^{*},R^{*}) = 0$. Moreover, $\frac{{dF}}{{dt}} = [ (S + E + I + A + R) - (S^{*} + E^{*} + I^{*} + A^{*} + R^{*}) ]\frac{{dN}}{{dt}}$.

Since

$$ S^{*} + E^{*} + I^{*} + A^{*} + R^{*} = \frac{\Lambda }{\varpi } $$

and

$$ \frac{{dN}}{{dt}} = \Lambda - \alpha _{3}N - \alpha _{5}(A + I), $$

we have

$$ \frac{{dF}}{{dt}} = \biggl( N - \frac{\Lambda }{\alpha _{3}} \biggr) \bigl( \Lambda - \alpha _{3}N - \alpha _{3}(A + I) \bigr) = - \frac{1}{\alpha _{3}} ( \Lambda - \alpha _{3}N )^{2} - ( \Lambda - \alpha _{3}N ) (A + I) \le 0. $$

Note that at the EEP we have $N \le \frac{\Lambda }{\alpha _{3}}$. Hence, it follows that $\frac{{dF}}{{dt}} \le 0$ and $\frac{{dF}}{{dt}} = 0$ if and only if $S = S^{*}$, $E = E^{*}$, $I = I^{*}$, $A = A^{*}$, $R = R^{*}$. Therefore the largest closed and bounded invariant set in $\{ S,E,I,A,R \in \Omega :\dot{F} = 0 \} $ is the set $\{ N^{*}:N^{*} = (S^{*},E^{*},I^{*},A^{*},R^{*}) \} $. By LaSalle’s invariance principle the unique equilibrium point $N^{*}$ is globally asymptotically stable when $R_{0} > 1$ in the region Ω. □

## Numerical solution of the model

In this section, we develop a numerical scheme for model (3b) using the Toufik–Atangana rule detailed in [27].

Now from the first equation of (3b) we have

18 $$ \begin{aligned} &{}_{0}^{{\mathrm{ABC}}}D_{t}^{\eta } S(t) = G_{1} \bigl(t,S(t) \bigr), \\ &S(0) = S_{0}. \end{aligned} $$

Based on (4), we obtain the solution for (18) given in (19):

19 $$ S(t) = S(0) + \frac{1 - \eta }{F(\eta )}G_{1} \bigl(t,S(t) \bigr) + \frac{\eta }{F(\eta )\Gamma (\eta )} \int _{0}^{t} G_{1} \bigl(k,S(k) \bigr) (t - k)^{\eta - 1}{\,dk}. $$

Applying Lagrange’s interpolation polynomial on the interval $[t_{k}, t_{k + 1}]$ to the equality $G_{1}(y,S(y)) = \frac{\Lambda }{\alpha _{3}} - b_{0}\frac{(I(y) + A(y))S(y)}{k(N)} - S(y)$ leads to

20 $$\begin{aligned} S_{K} \approx& \frac{1}{h} \bigl[ ( y - t_{k - 1} )G_{1} \bigl(t_{k},S(t_{k}),I(t_{k}),A(t_{k}) \bigr) \\ &{}- ( y - t_{k} )G_{1} \bigl(t_{k - 1},S(t_{k - 1}),I(t_{k - 1}),A(t_{k - 1}) \bigr) \bigr], \end{aligned}$$

where $h = t_{k} - t_{k - 1}$.

Now substituting (20) into (19), we have

21 $$\begin{aligned} S(t_{n + 1}) =& S(0) + \frac{1 - \eta }{F(\eta )}G_{1} \bigl(t_{k},S(t_{k}),I(t_{k}),A(t_{k}) \bigr) \\ &{}+ \frac{\eta }{F(\eta )\Gamma (\eta )}\sum_{j = 1}^{n} \left (\textstyle\begin{array}{l} \frac{G_{1}(t_{j},S(t_{j}),I(t_{j}),A(t_{j}))}{h}\int _{t_{j}}^{t_{j + 1}} ( y - t_{j - 1} ) (t_{n + 1} - y)^{\eta - 1}{\,dy} \\ \quad {}- \frac{G_{1}(t_{j - 1},S(t_{j - 1}),I(t_{j - 1}),A(t_{j - 1}))}{h}\int _{t_{j}}^{t_{j + 1}} ( y - t_{j - 1} ) (t_{n + 1} - y)^{\eta - 1}{\,dy} \end{array}\displaystyle \right ) \\ =& S(0) + \frac{1 - \eta }{F(\eta )}G_{1}\bigl(t_{n},S(t_{n}),I(t_{n}),A(t_{n}) \bigr) \\ &{}+ \frac{\eta }{F(\eta )\Gamma (\eta )}\sum_{j = 1}^{n} \biggl( \frac{G_{1}(t_{j},S(t_{j}),I(t_{j}),A(t_{j}))}{h}\Upsilon _{j - 1} \\ & {}- \frac{G_{1}(t_{j - 1},S(t_{j - 1}),I(t_{j - 1}),A(t_{j - 1}))}{h}\Upsilon _{j} \biggr), \end{aligned}$$

where

22 $$\begin{aligned}& \begin{aligned}[b] \Upsilon _{j - 1} &= \int _{t_{j}}^{t_{j + 1}} ( y - t_{j - 1} ) (t_{n + 1} - y)^{\eta - 1}{\,dy} \\ &= - \frac{1}{\eta } \bigl[ ( t_{j + 1} - t_{j - 1} ) ( t_{n + 1} - t_{j + 1} )^{\eta } - ( t_{j} - t_{j - 1} ) ( t_{n + 1} - t_{j} )^{\eta } \bigr] \\ &\quad {}- \frac{1}{\eta (\eta + 1)} \bigl[ ( t_{n + 1} - t_{j + 1} )^{\eta + 1} ( t_{n + 1} - t_{j + 1} )^{\eta } - ( t_{n + 1} - t_{j} )^{\eta + 1} \bigr], \end{aligned} \end{aligned}$$

23 $$\begin{aligned}& \begin{aligned}[b] \Upsilon _{j} &= \int _{t_{j}}^{t_{j + 1}} ( y - t_{j - 1} ) (t_{n + 1} - y)^{\eta - 1}{\,dy} \\ &= - \frac{1}{\eta } \bigl[ ( t_{j + 1} - t_{j - 1} ) ( t_{n + 1} - t_{j + 1} )^{\eta } \bigr] \\ &\quad {}- \frac{1}{\eta (\eta + 1)} \bigl[ ( t_{n + 1} - t_{j + 1} )^{\eta + 1} - ( t_{n + 1} - t_{j} )^{\eta + 1} \bigr]. \end{aligned} \end{aligned}$$

Furthermore, substituting $t_{j} = {jh}$ into (22) and (23) leads to

24 $$\begin{aligned}& \Upsilon _{j - 1} = \frac{h^{\eta + 1}}{\eta (\eta + 1)} \bigl[ ( n + 1 - j )^{\eta } ( n - j + 2 + \eta ) - ( n - j )^{\eta } ( n - j + 2 + 2\eta ) \bigr], \end{aligned}$$

25 $$\begin{aligned}& \Upsilon _{j} = \frac{h^{\eta + 1}}{\eta (\eta + 1)} \bigl[ ( n + 1 - j )^{\eta + 1} - ( n - j )^{\eta } (n - j + 1 + \eta ) \bigr]. \end{aligned}$$

Finally, we can express (21) in terms of (24) and (25) as follows:

26 $$\begin{aligned} S(t_{n + 1}) =& S(t_{0}) + \frac{1 - \eta }{F(\eta )}G_{1} \bigl(t_{n},S(t_{n}),I(t_{n}),A(t_{n}) \bigr)+ \frac{\eta }{F(\eta )\Gamma (\eta )} \\ &{}\times\sum_{j = 1}^{n} \left (\textstyle\begin{array}{l} ( \frac{G_{1}(t_{j},S(t_{j}),I(t_{j}),A(t_{j}))}{\Gamma (\eta + 2)} ) \\ \quad {}\times h^{\eta } [ ( n + 1 - j )^{\eta } ( n - j + 2 + \eta ) - ( n - j )^{\eta } ( n - j + 2 + 2\eta ) ] \\ \quad {}- ( \frac{G_{1}(t_{j - 1},S(t_{j - 1}),I(t_{j - 1}),A(t_{j - 1}))}{\Gamma (\eta + 2)} ) \\ \quad {}\times h^{\eta } [ ( n + 1 - j )^{\eta + 1} - ( n - j )^{\eta } (n - j + 1 + \eta ) ] \end{array}\displaystyle \right ). \end{aligned}$$

In the same way, we have the following equations for the remaining state variables:

27 $$\begin{aligned}& \begin{aligned}[b] E(t_{n + 1}) &= E(t_{0}) + \frac{1 - \eta }{F(\eta )}G_{2}\bigl(t_{n},S(t_{n}),E(t_{n}),I(t_{n}),A(t_{n}),R(t_{n}) \bigr)+ \frac{\eta }{F(\eta )\Gamma (\eta )} \\ &\quad {}\times\sum_{j = 1}^{n} \left (\textstyle\begin{array}{l} \frac{G_{2}(t_{j},S(t_{j}),E(t_{j}),I(t_{j}),A(t_{j}),R(t_{j})}{\Gamma (\eta + 2)} \\ \quad {}\times h^{\eta } [ ( n + 1 - j )^{\eta } ( n - j + 2 + \eta ) - ( n - j )^{\eta } ( n - j + 2 + 2\eta ) ] \\ \quad {}- \frac{G_{2}(t_{j - 1},S(t_{j - 1}),E(t_{j - 1}),I(t_{j - 1}),A(t_{j - 1}),R(t_{j - 1}))}{\Gamma (\eta + 2)} \\ \quad {}\times h^{\eta } [ ( n + 1 - j )^{\eta + 1} - ( n - j )^{\eta } (n - j + 1 + \eta ) ] \end{array}\displaystyle \right ), \end{aligned} \end{aligned}$$

28 $$\begin{aligned}& \begin{aligned}[b] A(t_{n + 1}) &= A(t_{0}) + \frac{1 - \eta }{F(\eta )}G_{3}\bigl(t_{n},S(t_{n}),E(t_{n}),I(t_{n}),A(t_{n}),R(t_{n}) \bigr)+ \frac{\eta }{F(\eta )\Gamma (\eta )} \\ &\quad {}\times\sum_{j = 1}^{n} \left (\textstyle\begin{array}{l} \frac{G_{3}(t_{j},S(t_{j}),E(t_{j}),I(t_{j}),A(t_{j}),R(t_{j})}{\Gamma (\eta + 2)} \\ \quad {}\times h^{\eta } [ ( n + 1 - j )^{\eta } ( n - j + 2 + \eta ) - ( n - j )^{\eta } ( n - j + 2 + 2\eta ) ] \\ \quad {}- \frac{G_{3}(t_{j - 1},S(t_{j - 1}),E(t_{j - 1}),I(t_{j - 1}),A(t_{j - 1}),R(t_{j - 1}))}{\Gamma (\eta + 2)} \\ \quad {}\times h^{\eta } [ ( n + 1 - j )^{\eta + 1} - ( n - j )^{\eta } (n - j + 1 + \eta ) ] \end{array}\displaystyle \right ), \end{aligned} \end{aligned}$$

29 $$\begin{aligned}& \begin{aligned}[b] I(t_{n + 1}) &= I(t_{0}) + \frac{1 - \eta }{F(\eta )}G_{4}\bigl(t_{n},S(t_{n}),E(t_{n}),I(t_{n}),A(t_{n}),R(t_{n}) \bigr)+ \frac{\eta }{F(\eta )\Gamma (\eta )} \\ &\quad {}\times\sum_{j = 1}^{n} \left (\textstyle\begin{array}{l} \frac{G_{4}(t_{j},S(t_{j}),E(t_{j}),I(t_{j}),A(t_{j}),R(t_{j})}{\Gamma (\eta + 2)} \\ \quad {}\times h^{\eta } [ ( n + 1 - j )^{\eta } ( n - j + 2 + \eta ) - ( n - j )^{\eta } ( n - j + 2 + 2\eta ) ] \\ \quad {}- \frac{G_{4}(t_{j - 1},S(t_{j - 1}),E(t_{j - 1}),I(t_{j - 1}),A(t_{j - 1}),R(t_{j - 1}))}{\Gamma (\eta + 2)} \\ \quad {}\times h^{\eta } [ ( n + 1 - j )^{\eta + 1} - ( n - j )^{\eta } (n - j + 1 + \eta ) ] \end{array}\displaystyle \right ), \end{aligned} \end{aligned}$$

30 $$\begin{aligned}& \begin{aligned}[b] R(t_{n + 1}) &= R(t_{0}) + \frac{1 - \eta }{F(\eta )}G_{5}\bigl(t_{n},S(t_{n}),E(t_{n}),I(t_{n}),A(t_{n}),R(t_{n}) \bigr)+ \frac{\eta }{F(\eta )\Gamma (\eta )} \\ &\quad {}\times\sum_{j = 1}^{n} \left (\textstyle\begin{array}{l} \frac{G_{5}(t_{j},S(t_{j}),E(t_{j}),I(t_{j}),A(t_{j}),R(t_{j})}{\Gamma (\eta + 2)} \\ \quad {}\times h^{\eta } [ ( n + 1 - j )^{\eta } ( n - j + 2 + \eta ) - ( n - j )^{\eta } ( n - j + 2 + 2\eta ) ] \\ \quad {}- \frac{G_{5}(t_{j - 1},S(t_{j - 1}),E(t_{j - 1}),I(t_{j - 1}),A(t_{j - 1}),R(t_{j - 1}))}{\Gamma (\eta + 2)} \\ \quad {}\times h^{\eta } [ ( n + 1 - j )^{\eta + 1} - ( n - j )^{\eta } (n - j + 1 + \eta ) ] \end{array}\displaystyle \right ). \end{aligned} \end{aligned}$$

### Numerical simulation I

The purpose of this section is to investigate the impact of different values of fractional order *η* in model (3b). To this end, we give several numerical simulations of the model using a numerical technique developed by Toufic and Atangana as shown in equations (26)–(30). We took some approximations to the real values of the parameters given as

$$\begin{aligned}& N = 1\text{,}000\text{,}000,\qquad \Lambda = 0.003N,\qquad \rho = 0.17,\qquad \alpha = 0.00037, \\& \alpha _{1} = 0.003,\qquad \alpha _{3} = 0.000037, \qquad \alpha _{4} = 0.0180322,\qquad \alpha _{5} = 0.0002, \\& \alpha _{6} = 0.19,\qquad \alpha _{7} = 0.00023, \qquad b_{0} = \alpha \alpha _{1},\qquad b = 0.02,\qquad h = 0.1. \end{aligned}$$

The hypothetical initial population used for the numerical simulation is

$$ (S_{0},E_{0},A_{0},I_{0},R_{0}) = (2\text{,}999\text{,}979,20,1,0,0). $$

The numerical simulations and corresponding descriptions are given below.

**Figure 2:**
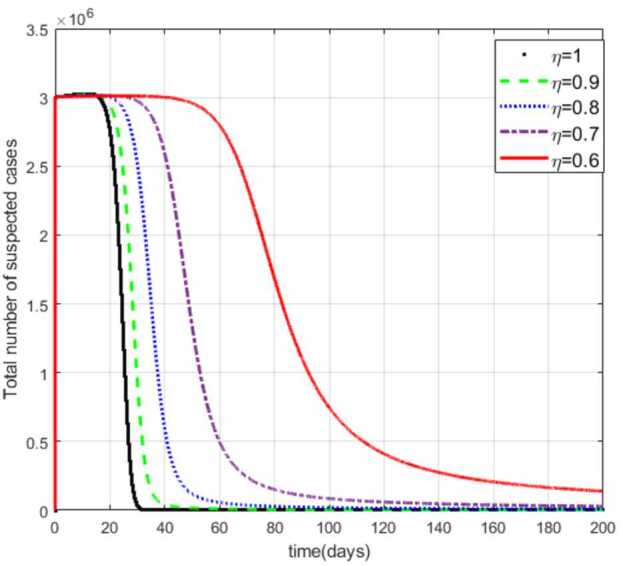
Total number of suspected cases (S) for different values of *η*

Figure 2 shows that decreasing the fractional order of derivative *η* from 1 leads to flattening the curves of the suspected cases (S) with a few decreases in the number of cases in the compartment. As the value *η* gets smaller and smaller, the number of cases approaches the constant value $S_{0}$.

**Figure 3:**
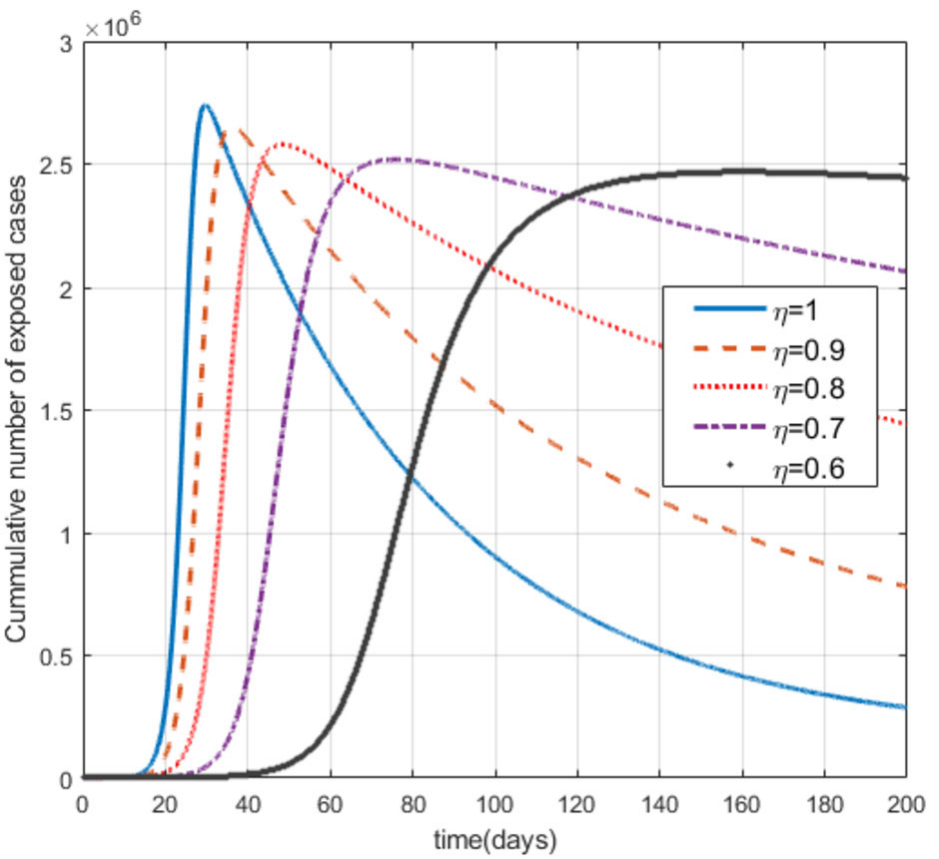
Total number of exposed (E) cases for different values of *η*

**Figure 4:**
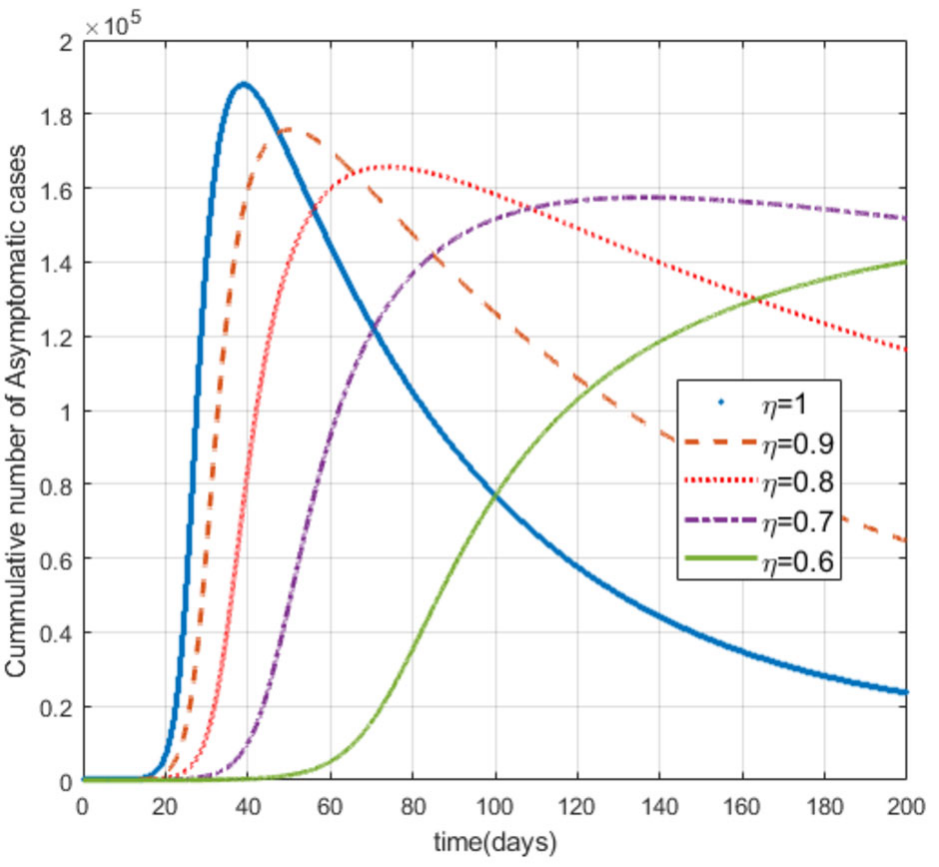
Total number of asymptomatic cases (A) for different values of *η*

**Figure 5:**
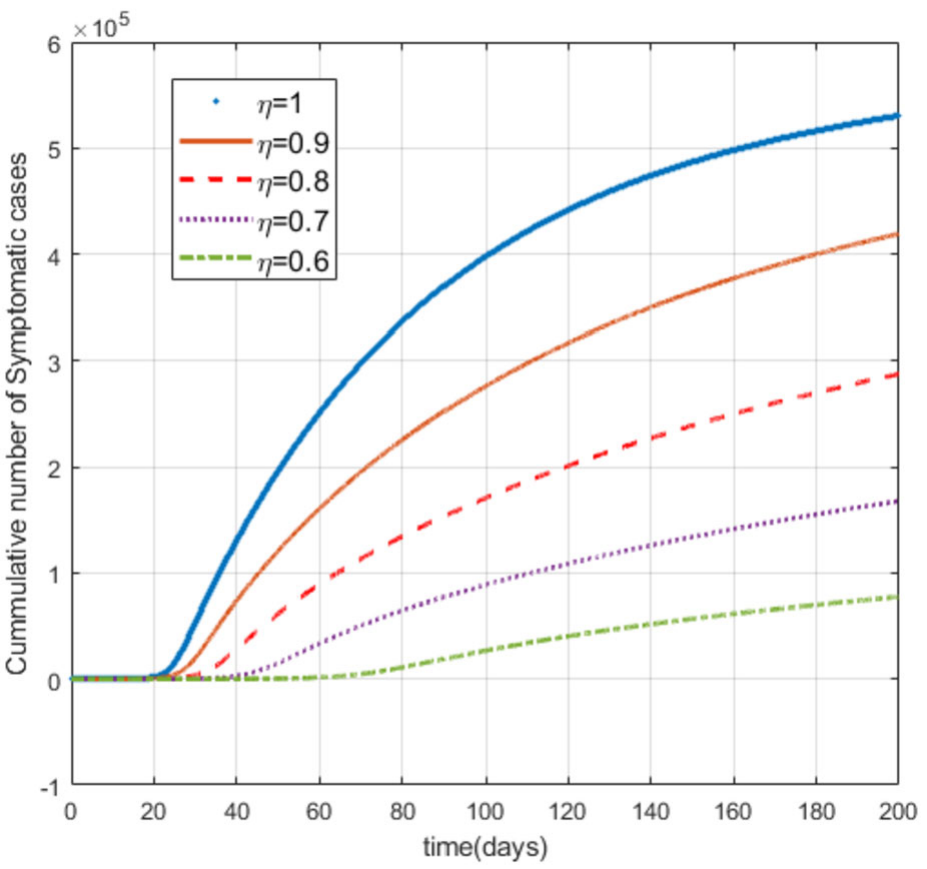
Total number of symptomatic cases (I) for different values of *η*

**Figure 6:**
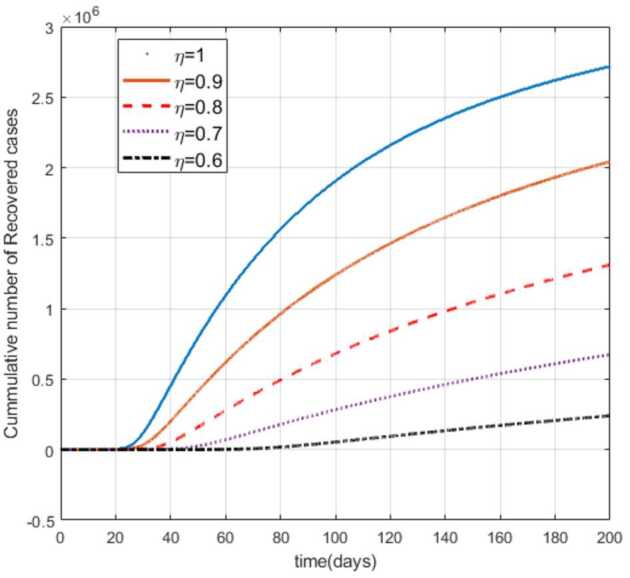
Total number of recovered cases (R) for different values of *η*

Moreover, from Figs. 3–6 we can say that decreasing the fractional order of derivative *η* leads to a decrease in the number of exposed, asymptotically infected, symptomatically infected, and recovered cases significantly. In other words, the curves for each of the compartments E, A, I, and R get flattened as *η* reduces from 1 to 0.6 as shown in the figures. We can conclude that as *η* gets close to zero from the right, the number of cases in E, A, I, and R gets close to zero. It must also be noted that the EEP for fractional order and the integer order are the same and the solution of the fractional order tends to the EEP over a longer time. From Figs. 2–6 it seems that as the fractional derivative gets smaller and smaller, the time it requires to approach to the EEP gets longer and longer. That is, when the derivative order is reduced from 1, the memory effect of the dynamic system increases, and, consequently, the infection in each of the compartments increases slowly for a long time. Hence the fractional derivative order *η* affects the dynamics of infection of model (3b) at least in this work. The peak period of the compartments E, A, R, and I is prolonged, and the number of cases at the peak is reduced as *η* reduces from 1 to zero (see Figs. 3–6).

## Optimal control analysis

We consider a generalized form of (3b) by including the control variable $u(t)$, which ranges from 0 to 1, where zero corresponds to the absence of application of any control mechanism, and one refers to the case where there is a fully controlled scenario, which is nearly impossible to attain in many endemic disease cases such as COVID-19. The intermediate values $u(t) \in (0,1)$ quantify the effect of applying the intervention mechanisms. In this model, the purpose of incorporating the control variable is reducing the number of exposed, asymptomatic, and symptomatic individuals by a factor of $( 1 - u )$. Model (3b) is modified to (31) after incorporating the control variable:

31 $$ \left . \textstyle\begin{array}{l} {}_{0}^{{\mathrm{ABC}}}D_{t}^{\eta } S(t) = \Lambda - ( 1 - u )b_{0}\frac{(I + A)S}{k(N)} - \alpha _{3}S, \\ {}_{0}^{{\mathrm{ABC}}}D_{t}^{\eta } E(t) = ( 1 - u )b_{0}\frac{(I + A)S}{k(N)} - ( \alpha _{3} + \alpha _{4} )E, \\ {}_{0}^{{\mathrm{ABC}}}D_{t}^{\eta } A(t) = (1 - \rho )\alpha _{4}E - ( \alpha _{3} + \alpha _{5} + \alpha _{6} )A, \\ {}_{0}^{{\mathrm{ABC}}}D_{t}^{\eta } I(t) = \rho \alpha _{4}E - ( \alpha _{3} + \alpha _{5} + \alpha _{7} )I, \\ {}_{0}^{{\mathrm{ABC}}}D_{t}^{\eta } R(t) = \alpha _{6}A + \alpha _{7}I - \alpha _{3}R, \end{array}\displaystyle \right \} $$

and the main objective is to find the control optimal unit $u(t)$ such that the following control objective function is minimized:

32 $$ J_{o}(u) = \int _{0}^{T} \biggl( {ab}_{0} \frac{(I + A)S}{k(N)} + \frac{b}{2}u^{2}(t) \biggr){\, dt}, $$

where *a* is a nonnegative weight for the endemic diseases, and *b* measures the relative cost of interventions over the range of $[0,T]$.

The objective is minimizing the number of exposed, symptomatically, or asymptomatically infected cases while minimizing the cost of control $u(t):[0,T] \to [0,1]$.

To solve (31) and (32), we derive the necessary optimality conditions for the problem. To do this, we define the scaler Hamiltonian function

33 $$\begin{aligned}& H\bigl(y,u(t),\lambda _{j},t\bigr) \\& \quad = {ab}_{0}\frac{(I + A)S}{k(N)} + \frac{b}{2}u^{2}(t) + \lambda _{S}h_{1}(t) + \lambda _{E}h_{2}(t) + \lambda _{A}h_{3}(t) + \lambda _{I}h_{4}(t) + \lambda _{R}h_{6}(t), \end{aligned}$$

where

$$\begin{aligned}& h_{1}(t) = \Lambda - ( 1 - u )b_{0} \frac{(I + A)S}{k(N)} - \alpha _{3}S, \\& h_{2}(t) = ( 1 - u )b_{0}\frac{(I + A)S}{k(N)} - ( \alpha _{3} + \alpha _{4} )E, \\& h_{3}(t) = (1 - \rho )\alpha _{4}E - ( \alpha _{3} + \alpha _{5} + \alpha _{6} )A, \\& h_{4}(t) = \rho \alpha _{4}E - ( \alpha _{3} + \alpha _{5} + \alpha _{7} )I, \\& h_{5}(t) = \alpha _{6}A + \alpha _{7}I - \alpha _{3}R, \end{aligned}$$

and

$$\begin{aligned}& \lambda _{S},\lambda _{E},\lambda _{A}, \lambda _{I},\lambda _{R} \mbox{ are the adjoint variables}, \\& \lambda _{j} = (\lambda _{S},\lambda _{E},\lambda _{A},\lambda _{I},\lambda _{R} ), \\& y = (S,E,A,I,R). \end{aligned}$$

Following [14], in equations (34)–(37), we obtain the necessary optimality conditions for the system of equations (31) and (32):

34 $$\begin{aligned}& \left . \textstyle\begin{array}{l} {}_{0}^{{\mathrm{ABC}}}D_{t}^{\eta } S(t) = \frac{\partial H}{\partial \lambda _{S}}(t), \qquad {}_{0}^{{\mathrm{ABC}}}D_{t}^{\eta } E(t) = \frac{\partial H}{\partial \lambda _{E}}(t), \\ {}_{0}^{{\mathrm{ABC}}}D_{t}^{\eta } A(t) = \frac{\partial H}{\partial \lambda _{A}}(t),\qquad {}_{0}^{{\mathrm{ABC}}}D_{t}^{\eta } I(t) = \frac{\partial H}{\partial \lambda _{I}}(t), \\ {}_{0}^{{\mathrm{ABC}}}D_{t}^{\eta } R(t) = \frac{\partial H}{\partial \lambda _{R}}(t), \end{array}\displaystyle \right \} \end{aligned}$$

35 $$\begin{aligned}& \left . \textstyle\begin{array}{l} {}_{t}^{{\mathrm{ABC}}}D_{T}^{\eta } \lambda _{S}(t) = - \frac{\partial H}{\partial S}(t),\qquad {}_{t}^{{\mathrm{ABC}}}D_{T}^{\eta } \lambda _{E}(t) = - \frac{\partial H}{\partial E}(t), \\ {}_{t}^{{\mathrm{ABC}}}D_{T}^{\eta } \lambda _{A}(t) = - \frac{\partial H}{\partial A}(t),\qquad {}_{t}^{{\mathrm{ABC}}}D_{T}^{\eta } \lambda _{I}(t) = - \frac{\partial H}{\partial I}(t), \\ {}_{t}^{{\mathrm{ABC}}}D_{T}^{\eta } \lambda _{R}(t) = - \frac{\partial H}{\partial R}(t), \end{array}\displaystyle \right \} \end{aligned}$$

36 $$\begin{aligned}& \frac{\partial H}{\partial u}(t) = 0, \end{aligned}$$

with transversality conditions

37 $$ \lambda _{j}(T) = 0,\quad j \in \{ \lambda _{S}, \lambda _{E},\lambda _{A},\lambda _{I}, \lambda _{R}\}. $$

Solving the system of equations (34) is the same as solving (31), and solving the system of equations (35) is the same as solving the system of adjoint equations given by

38 $$ \left . \textstyle\begin{array}{l} {}_{t}^{{\mathrm{ABC}}}D_{T}^{\eta } \lambda _{S}(t) = - \frac{{ab}_{0}(A + I)}{k(N)} + \lambda _{S} ( \alpha _{3} - \frac{(b_{0}(u - 1)(A + I)}{k(N)} ) + \frac{b_{0}\lambda _{E}(u - 1)(A + I)}{k(N)}, \\ {}_{t}^{{\mathrm{ABC}}}D_{T}^{\eta } \lambda _{E}(t) = - \lambda _{A} + \lambda _{E}(\alpha _{3} + \alpha _{4}) + \alpha _{4}\rho \lambda _{I}, \\ {}_{t}^{{\mathrm{ABC}}}D_{T}^{\eta } \lambda _{A}(t) = - \alpha _{6}\lambda _{R} + \lambda _{A}( \alpha _{3} + \alpha _{5} + \alpha _{6}) - \frac{{ab}_{0}S}{k(N)} - \frac{b_{0}S(u - 1)\lambda _{E}}{k(N)} + \frac{b_{0}S(u - 1)\lambda _{S}}{k(N)}, \\ {}_{t}^{{\mathrm{ABC}}}D_{T}^{\eta } \lambda _{I}(t) = - \alpha _{7}\lambda _{R} + \lambda _{I}(\alpha _{3} + \alpha _{5} + \alpha _{7}) - \frac{{ab}_{0}S}{k(N)} + \frac{b_{0}S(u - 1)\lambda _{E}}{k(N)} - \frac{b_{0}S(u - 1)\lambda _{S}}{k(N)}, \\ {}_{t}^{{\mathrm{ABC}}}D_{T}^{\eta } \lambda _{R}(t) = \alpha _{3}\lambda _{R}. \end{array}\displaystyle \right \} $$

Accordingly, the optimal control $u*(t)$ of a dynamic system (31), which minimizes the objective functional (32), is characterized by

39 $$ u*(t) = \min \biggl[ \max \biggl( 0,\frac{b_{0}S(A + I)}{{bk}(N)}(\lambda _{E} - \lambda _{S}) \biggr),1 \biggr]. $$

Moreover, the fractional derivative state equation (31), the fractional derivative adjoint equation (38), together with the characterization of the optimal control (39) and the boundary conditions (37), are the optimality systems. The aforementioned equations of the optimality system represent the analytic solution of the optimal control system under discussion. In the next sub-section, we consider the numerical solutions and simulations.

### Numerical simulation II

In this section, we simulate the optimality system given by (31), (38), (37), and (39) using the Toufik–Atangana numerical scheme and a computational software Matlab2018a. We apply the numerical scheme to these equations in a similar way we applied the method to (3b) in Sect. 5.1. The initial conditions and the parameter values used are the same as those in Sect. 5.1.

**Figure 7:**
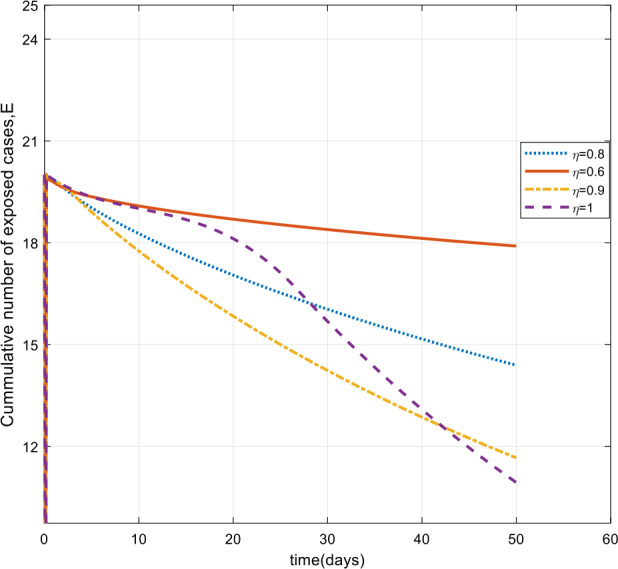
Number of exposed cases (E) with control for different values of the fractional derivative order *η*

**Figure 8:**
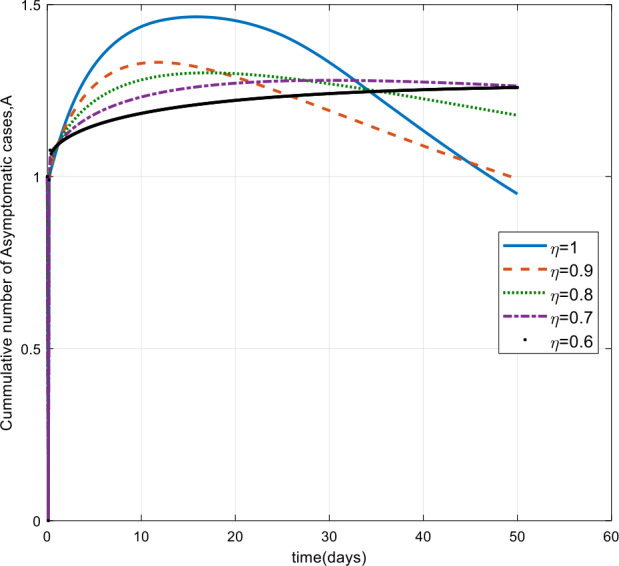
Number of asymptomatic cases (A) with control for different values of the fractional derivative order *η*

**Figure 9:**
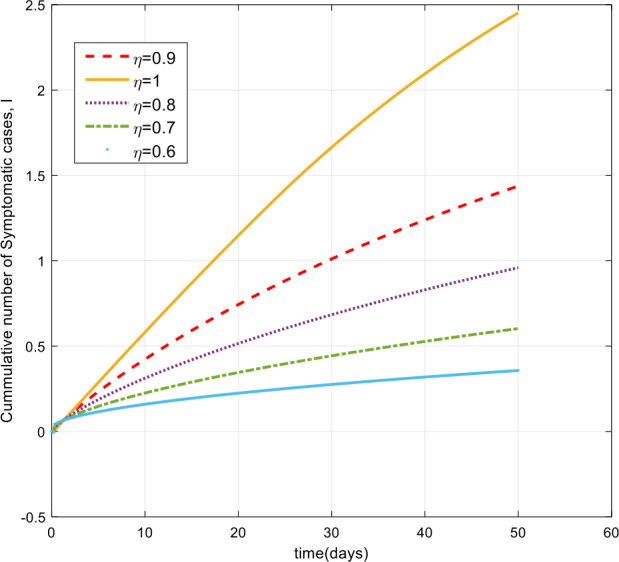
Number of symptomatic cases (I) with the control for different values of the fractional derivative order *η*

**Figure 10:**
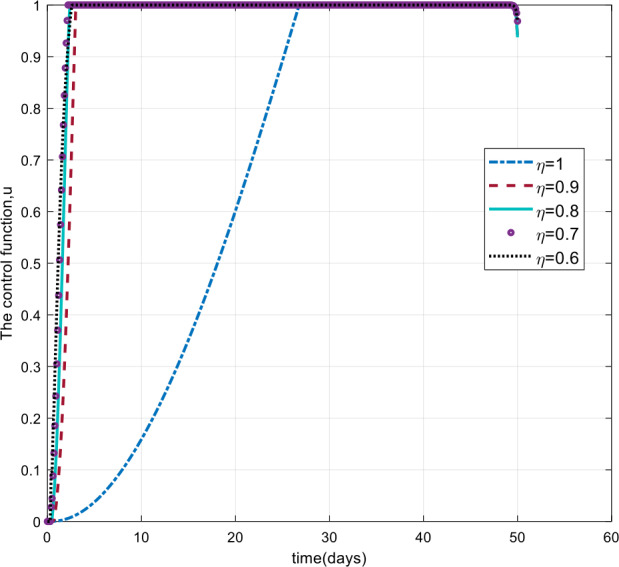
The control profile $u(t)$ for different values of the fractional derivative order *η*

As can be seen from Figs. 7, 8, and 9, the number of cases in the compartments of E, A, and I, declined significantly as compared to Figs. 3–5, which are simulated without application of the control strategy. However, the curves exhibit different asymptotic behaviors for different values of the fractional derivative *η*. The difference in the asymptotic behavior of the curves for different fractional orders is also observed in the optimal profile of the control function depicted in Fig. 10 for different values of *η*. The decrease in the number of cases in E, A, and I after applying the control strategy verifies the efficiency of the proposed optimal control strategy.

### Conclusions

In this work, we studied a SEAIR epidemic mathematical model involving the Atangana–Baleanu fractional derivative. The result of the simulation shows that the reduction of the order of the fractional derivative from 1 resulted in flattening of the curves, and the endemic decreases slowly for the suspected (S) cases (Fig. 2). The curves for the compartments E, A, I, and R show that in each of them, as the fractional order decreases from 1, the spread of the endemic grows slowly, and the number of cases at peak gets relatively smaller and smaller (Figs. 3–6).

It is of utmost importance to decrease the number of cases in the different compartments of an endemic model, and the result of this study showed that the decrease in the number of cases happens as the fractional order decreases. This important result is attributed to the Atangana–Baleanu fractional operator with the hereditary property. We, the authors of this paper, claim that the hidden or actual properties of real-world phenomena can be revealed better by mathematical models involving the Atangana–Baleanu fractional operator. This argument can be substantiated by conducting further studies on the effect of other fractional operators such as Caputo–Fabrizio fractional derivatives and comparing the results with the Atangana–Baleanu fractional operator result on the same model used or other pertinent epidemic models. In this study the absence of comparing the result obtained in this study for the SEAIR model by using the Atangana–Baleanu fractional operator with results that could be obtained by applying the other fractional operators for the same model can be a gap for future work.

## Data Availability

Not applicable.

## References

[1] Dietz K., Heesterbeek J.A. (2000). Bernoulli was ahead of modern epidemiology. Nature.

[2] Kermack W.O., McKendrick A.G. (1991). Contributions to the mathematical theory of epidemics—I. Bull. Math. Biol..

[3] Zhang J., Ma Z. (2003). Global dynamics of an SEIR epidemic model with saturating contact rate. Math. Biosci..

[4] Li X.Z., Zhou L.L. (2009). Global stability of an SEIR epidemic model with vertical transmission and saturating contact rate. Chaos Solitons Fractals.

[5] Deressa C.T., Duressa G.F. (2021). Modeling and optimal control analysis of transmission dynamics of COVID-19: the case of Ethiopia. Alex. Eng. J..

[6] Deressa C.T., Mussa Y.O., Duressa G.F. (2020). Optimal control and sensitivity analysis for transmission dynamics of coronavirus. Results Phys..

[7] Xu R., Ma Z., Wang Z. (2010). Global stability of a delayed SIRS epidemic model with saturation incidence and temporary immunity. Comput. Math. Appl..

[8] Sene N. (2020). Analysis of the stochastic model for predicting the novel coronavirus disease. Adv. Differ. Equ..

[9] Borah M.J., Hazarika B., Panda S.K., Nieto J.J. (2020). Examining the correlation between the weather conditions and COVID-19 pandemic in India: a mathematical evidence. Results Phys..

[10] Khan H., Gómez-Aguilar J.F., Alkhazzan A., Khan A. (2020). A fractional order HIV-TB coinfection model with nonsingular Mittag-Leffler law. Math. Methods Appl. Sci..

[11] Das S. (2011). Functional Fractional Calculus.

[12] Ross B. (1977). The development of fractional calculus 1695–1900. Hist. Math..

[13] Atangana A. (2020). Extension of rate of change concept: from local to nonlocal operators with applications. Results Phys..

[14] Baleanu D., Jajarmi A., Sajjadi S.S., Mozyrska D. (2019). A new fractional model and optimal control of a tumor-immune surveillance with non-singular derivative operator. Chaos, Interdiscip. J. Nonlinear Sci..

[15] Sene N. (2020). Fractional diffusion equation with new fractional operator. Alex. Eng. J..

[16] Sene N., Sène B., Ndiaye S.N., Traoré A. (2020). Novel approaches for getting the solution of the fractional Black–Scholes equation described by Mittag-Leffler fractional derivative. Discrete Dyn. Nat. Soc..

[17] Sene N. (2020). Analysis of a four-dimensional hyperchaotic system described by the Caputo–Liouville fractional derivative. Complexity.

[18] Khan M.A., Atangana A., Alzahrani E. (2020). The dynamics of COVID-19 with quarantined and isolation. Adv. Differ. Equ..

[19] Ghanbari B., Atangana A. (2020). Some new edge detecting techniques based on fractional derivatives with non-local and non-singular kernels. Adv. Differ. Equ..

[20] Panda S.K., Ravichandran C., Hazarika B. (2021). Results on system of Atangana–Baleanu fractional order Willis aneurysm and nonlinear singularly perturbed boundary value problems. Chaos Solitons Fractals.

[21] Panda S.K. (2020). Applying fixed point methods and fractional operators in the modelling of novel coronavirus 2019-nCoV/SARS-CoV-2. Results Phys..

[22] Zhang Z. (2020). A novel COVID-19 mathematical model with fractional derivatives: singular and nonsingular kernels. Chaos Solitons Fractals.

[23] Din A., Shah K., Seadawy A., Alrabaiah H., Baleanu D. (2020). On a new conceptual mathematical model dealing the current novel coronavirus-19 infectious disease. Results Phys..

[24] Qureshi S., Atangana A. (2020). Fractal-fractional differentiation for the modeling and mathematical analysis of nonlinear diarrhea transmission dynamics under the use of real data. Chaos Solitons Fractals.

[25] Atangana A., Araz Sİ. (2020). New numerical method for ordinary differential equations: Newton polynomial. J. Comput. Appl. Math..

[26] Khan M.A., Atangana A. (2020). Modeling the dynamics of novel coronavirus (2019-nCov) with fractional derivative. Alex. Eng. J..

[27] Toufik M., Atangana A. (2017). New numerical approximation of fractional derivative with non-local and non-singular kernel: application to chaotic models. Eur. Phys. J. Plus.

[28] Ghandehari M.A., Ranjbar M. (2013). A numerical method for solving a fractional partial differential equation through converting it into an NLP problem. Comput. Math. Appl..

[29] Djida J.D., Atangana A., Area I. (2017). Numerical computation of a fractional derivative with non-local and non-singular kernel. Math. Model. Nat. Phenom..

[30] Owolabi K.M. (2018). Analysis and numerical simulation of multicomponent system with Atangana–Baleanu fractional derivative. Chaos Solitons Fractals.

[31] Demirci E., Unal A., Ozalp N. (2011). A fractional order SEIR model with density dependent death rate. Hacet. J. Math. Stat..

[32] Uçar S. (2020). Analysis of a basic SEIRA model with Atangana–Baleanu derivative. AIMS Math..

[33] Atangana, A., Baleanu, D.: New fractional derivatives with nonlocal and non-singular kernel: theory and application to heat transfer model. arXiv preprint (2016). arXiv:1602.03408

[34] Abdeljawad T., Baleanu D. (2017). Integration by parts and its applications of a new nonlocal fractional derivative with Mittag-Leffler nonsingular kernel. J. Nonlinear Sci. Appl..

[35] Singh J., Kumar D., Baleanu D. (2018). On the analysis of fractional diabetes model with exponential law. Adv. Differ. Equ..

[36] Dietz K. (1982). Overall population patterns in the transmission cycle of infectious disease agents. Population Biology of Infectious Disease.

[37] Odibat Z.M., Shawagfeh N.T. (2007). Generalized Taylor’s formula. Appl. Math. Comput..

[38] Baleanu D., Fernandez A. (2018). On some new properties of fractional derivatives with Mittag-Leffler kernel. Commun. Nonlinear Sci. Numer. Simul..

[39] Diekmann O., Heesterbeek J.A., Metz J.A. (1990). On the definition and the computation of the basic reproduction ratio $R_{0}$ in models for infectious diseases in heterogeneous populations. J. Math. Biol..

[40] Hale J.K. (1969). Ordinary Differential Equations.

